# γ-secretase facilitates retromer-mediated retrograde transport

**DOI:** 10.1242/jcs.263538

**Published:** 2025-02-20

**Authors:** Yuka Takeo, Mac Crite, Kashif Mehmood, Daniel DiMaio

**Affiliations:** ^1^ Department of Genetics, Yale School of Medicine, New Haven, CT 06510, USA; ^2^ Department of Molecular Biophysics and Biochemistry, Yale University, New Haven, CT 06510, USA; ^3^ Department of Therapeutic Radiology, Yale School of Medicine, New Haven, CT 06510, USA; ^4^ Yale Cancer Center, Yale School of Medicine, New Haven, CT 06510, USA

**Keywords:** Retromer, γ-secretase, Retrograde, Protein trafficking, Shiga toxin, Alzheimer disease

## Abstract

Retromer mediates retrograde transport of protein cargoes from endosomes to the *trans*-Golgi network (TGN). γ-secretase is a protease that cleaves the transmembrane domain of its target proteins. Although retromer can form a stable complex with γ-secretase, the functional consequences of this interaction are not known. Here, we report that retromer-mediated retrograde protein trafficking in cultured human epithelial cells is impaired by the γ-secretase inhibitor XXI or by knockout of PS1 (also known as PSEN1), the catalytic subunit of γ-secretase. These treatments inhibited endosome-to-TGN trafficking of retromer-dependent retrograde cellular cargoes, divalent metal transporter 1 isoform II, cation-independent mannose-6-phosphate receptor and shiga toxin, whereas trafficking of retromer-independent cargoes, cholera toxin and a mutant CIMPR unable to bind retromer was not affected. Moreover, we found that γ-secretase associates with retromer cargoes even in the absence of retromer. XXI treatment and PS1 knockout did not inhibit the ability of retromer or γ-secretase to associate with cargo and did not affect the expression of retromer subunits or Rab7-GTP, which regulates retromer–cargo interaction. These results imply that the γ-secretase–retromer interaction facilitates retromer-mediated retrograde trafficking of cellular transmembrane proteins.

## Teaser

**Highlighted Article:** γ-secretase associates with retromer, a complex involved in retrograde protein trafficking, and its cargo proteins. γ-secretase facilitates trafficking of retromer cargoes from the endosome to the *trans*-Golgi network.

## INTRODUCTION

Intracellular trafficking of proteins is under tight control to ensure proteins reach their proper destinations. Retromer is a key factor that initiates retrograde trafficking of cellular transmembrane (TM) proteins from the endosome to the *trans*-Golgi network (TGN), whereas γ-secretase is a transmembrane protease that plays no previously known role in retrograde trafficking of cellular proteins (Burd and Cullen, 2014).

Retromer is a highly conserved cytoplasmic protein complex that ensures the proper localization, sorting and recycling of certain TM proteins by regulating both retrograde transport of protein cargo from endosomes to the TGN, as well as anterograde transport of proteins from endosomes to the plasma membrane (Burd and Cullen, 2014; Seaman, 2021; Yoshida and Hasegawa, 2022). In mammals, retromer is composed of two major subcomplexes – a core cargo recognition trimer of vacuolar protein sorting-associated proteins 26, 29 and 35 [VPS26 (which has VPS26A–VPS26C forms in mammals), VPS29 and VPS35], which binds directly to protein cargo, and acts in concert with the second subcomplex, a membrane-associated sorting nexin (SNX) dimer (Seaman et al., 1997; Seaman, 2021). The large family of SNXs provide diversity in terms of cargo recognition and downstream trafficking pathways. Three distinct forms of the retromer complex are currently recognized – SNX-Bin, amphiphysin and Rvs-retromer (SNX-BAR-retromer), SNX3-retromer and SNX27-retromer (Attar and Cullen, 2010; Gallon and Cullen, 2015). Rab7 (herein referring to the Rab7a and Rab7b forms collectively unless otherwise indicated) is a small GTPase that regulates intracellular protein trafficking by cycling between a membrane-associated GTP-bound form and a cytosolic GDP-bound form (Guerra and Bucci, 2016; Stroupe, 2018). When bound to GTP, Rab7 recruits the retromer complex to the endosome membrane with its TM cargo and SNX proteins (Harrison et al., 2014; Rojas et al., 2008). The retromer–SNX complex induces tubulation of the endosome membrane, leading to the formation of a vesicle containing cargo that is transported to the TGN, where membrane fusion delivers the cargo to the TGN membrane (Purushothaman et al., 2017). The GTPase-activating protein (GAP), TBC1D5, stimulates generation of GDP-Rab7 and the dissociation of retromer and its cargo from the endosome membrane during trafficking (Xie et al., 2020).

Cellular retromer cargo proteins are TM proteins that typically possess a short sorting signal in their cytoplasmic domain that binds directly with the retromer for proper transport (Seaman, 2012). Cation-independent mannose 6-phosphate receptor (CIMPR; also known as IGF2R) and divalent metal transporter 1 (DMT1; also known as SLC11A2) isoform II (DMT1-II) are well-studied cellular proteins that are transported in a retrograde direction from the endosome to the TGN by retromer. DMT1-II plays a role in the efficient and rapid uptake of iron across the endosomal membrane in the transferrin cycle. SNX3 binds retromer at the interface of VPS35 and VPS26, causing a conformational change in VPS26, which allows recognition of the cytoplasmic domain of DMT1-II by VPS26 and SNX3 (Lucas et al., 2016). CIMPR associates with lysosomal enzymes and is transported from the Golgi to endosomes via vesicle transport. Subsequently, CIMPR is returned to the TGN by retrograde transport for reuse (Seaman, 2007). The role of retromer in the retrograde sorting of the CIMPR is complex. Although retromer bound to SNX proteins can bind to and transport CIMPR, SNX-BARs can also directly bind CIMPR independently of retromer and deliver CIMPR into the retrograde transport pathway (Shortill et al., 2022; Simonetti et al., 2017). Shiga toxin B-subunit (STxB) is a bacterial protein that is transported in a retrograde fashion when it enters cells, a process that requires a membrane-bound coat containing SNX1 and retromer (Bujny et al., 2007; Popoff et al., 2007; Sandvig and van Deurs, 2002).

γ-secretase is a protease complex composed of four subunits – presenilin 1 (PS1; also known as PSEN1), nicastrin (NCT; also known as NCSTN), anterior pharynx defective 1 (APH1, which has APH1A and APH1B forms in mammals) and presenilin enhancer 2 (PEN2; also known as PSENEN), each of which contains at least one TM domain (TMD) (Edbauer et al., 2003; Kimberly et al., 2003; Takasugi et al., 2003). γ-secretase specifically recognizes and cleaves substrate proteins, such as Notch and the amyloid precursor protein (APP) within their TMD (Yang et al., 2019; Zhou et al., 2019). In addition to its action as a protease, various non-proteolytic activities of γ-secretase have been described, such as acting as a scaffold to assemble protein complexes or as a chaperone to promote protein insertion into membranes (Inoue et al., 2018; Otto et al., 2016). Alzheimer disease (AD) is a neurodegenerative disease associated with the improper cleavage of APP by γ-secretase, leading to increased production of the amyloidogenic Aβ-42 peptide (Selkoe, 1998). Mutations within all four subunits of the γ-secretase complex can cause AD, with the majority of these mutations found in PS1, the catalytic subunit (Kelleher and Shen, 2017). Retromer dysfunction has also been implicated in AD and Parkinson disease (PD) pathogenesis (Qureshi et al., 2022; Siegenthaler and Rajendran, 2012; Small et al., 2005; Sullivan et al., 2011; Vilariño-Güell et al., 2011; Wen et al., 2011; Zhang et al., 2018a; Zimprich et al., 2011). For example, reduced expression of both VPS35 and VPS26 has been observed in the postmortem brains of individuals with AD, and mutations in VPS35, VPS29 and VPS26 are associated with the development of PD.

Although both γ-secretase and retromer are involved in neurodegeneration, it is not known whether γ-secretase affects retromer function. A stable complex between retromer and γ-secretase can be detected by co-immunoprecipitation from detergent extracts of murine neuronal cells, but the consequence of this interaction is not known (Ueda et al., 2016). Moreover, both γ-secretase and APP are recycled via retrograde trafficking, but it remains unclear whether their recycling depends on retromer (Kanatsu et al., 2018). In addition, both retromer and γ-secretase are required for delivery of human papillomaviruses (HPVs) into the retrograde trafficking pathway during virus entry into cells (Inoue et al., 2018; Lipovsky et al., 2013; Popa et al., 2015; Zhang et al., 2014, 2018b). However, delivery of HPV to the retrograde pathway is markedly different from retrograde trafficking of classical cellular cargoes. As noted above, cellular retromer cargoes are TM proteins stably integrated into the endosome membrane in the absence of retromer or γ-secretase function, whereas retrograde transport of HPV is triggered by γ-secretase-mediated insertion of a viral capsid protein into the endosome membrane, which is then stabilized by retromer (Inoue et al., 2018; Lipovsky et al., 2013; Popa et al., 2015; Zhang et al., 2014). It does not appear that either of these biochemical steps in HPV entry have a counterpart during retrograde trafficking of cellular cargo. Furthermore, it is not known whether retromer and γ-secretase cooperate during HPV entry or whether they play independent roles in this process. Because the functional relationship between γ-secretase and retromer, if any, is not known, we decided to test whether γ-secretase is required for the retromer-mediated retrograde trafficking of cellular cargo in uninfected cells.

In this study, we show that inhibition of γ-secretase activity and PS1 knockout (KO) cause accumulation of three different retromer cargoes in the endosome and their depletion from the TGN. Furthermore, we demonstrate that γ-secretase associates with retromer cargoes even in the absence of retromer. Inhibition of trafficking upon loss of γ-secretase does not appear to be due to reduced expression of the core retromer subunits, relocalization of retromer, inhibition of the association of the cargo with retromer or with γ-secretase, or perturbations in the expression or nucleotide loading of Rab7. Our findings suggest that γ-secretase supports retrograde trafficking of cellular protein cargoes by interacting with retromer and modulating retromer activity.

## RESULTS

### γ-secretase is required for efficient retromer-mediated trafficking of cellular protein cargoes

**Fig. 1.**
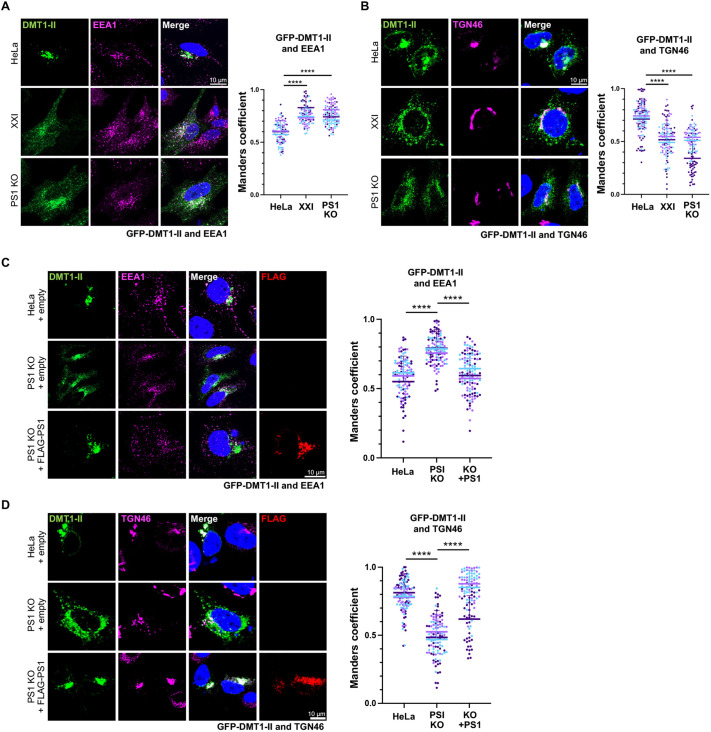
**γ-secretase inhibition and PS1 knockout inhibit retrograde trafficking of DMT1-II.** (A) HeLa control cells (HeLa) and PS1-knockout HeLa cells (PS1 KO) were treated with DMSO or 1 μM γ-secretase inhibitor XXI for 30 min and then transfected with a plasmid expressing GFP–DMT1-II. Cells were fixed 24 hpt and stained with DAPI and an antibody recognizing EEA1. Fluorescence images of single confocal planes are shown: GFP–DMT1-II, intrinsic green fluorescence; EEA1, magenta; nuclei, blue. Merged images show overlap between GFP–DMT1-II and EEA1 pseudocolored white. Graph shows Manders coefficients for colocalization of GFP–DMT1-II and EEA1 in cells expressing GFP–DMT1-II. Each dot represents the fluorescence intensity of an individual cell (with at least 30 cells counted per condition in each experiment); different colors are used to represent independent biological replicates. Horizonal lines and error bars indicate the mean±s.d. of the analyzed population in the three replicate experiments. Statistical significance was assessed after aggregating all three replicates for each condition. ****P<0.0001 (one-way ANOVA followed by a Tukey's multiple comparisons test). Statistical analysis of the means for this and other experiments is shown in Fig. S1. (B) As in A, except cells were stained with an antibody recognizing TGN46 instead of EEA1, and merged image shows overlap between GFP–DMT1-II and TNG46. (C) HeLa control and PS1 KO cells were transfected with a plasmid expressing GFP–DMT1-II and co-transfected with the empty control plasmid or a plasmid expressing FLAG–PS1. Cells were fixed 24 hpt and stained with DAPI and antibodies recognizing EEA1 and FLAG. Fluorescence images of single confocal planes are shown: GFP–DMT1-II, intrinsic green fluorescence; EEA1, magenta; FLAG–PS1, red; nuclei, blue. Merged images show overlap between GFP–DMT1-II and EEA1 pseudocolored white. Graph shows Manders coefficients for colocalization of GFP–DMT1-II and EEA1 in cells expressing GFP–DMT1-II [or in cells co-expressing GFP–DMT1-II and FLAG–PS1 in the case of cells transfected with plasmid expressing FLAG-PS1 (KO+FLAG-PS1)]. The graph and statistical analysis are as in A. (D) As in C, except cells were stained with antibodies recognizing TGN46 and FLAG, and merged images show overlap between GFP–DMT1-II and TGN46.

To determine whether γ-secretase plays a role in retromer-mediated trafficking of cellular proteins, we used immunofluorescence to monitor the effect of γ-secretase inhibition on DMT1-II trafficking. PS1-knockout (KO) HeLa cells, HeLa control cells and control cells treated with the specific γ-secretase inhibitor XXI were transfected with a plasmid expressing GFP-tagged DMT1-II. As assessed by immunofluorescence and confocal microscopy at 24 h post transfection (hpt), XXI treatment and PS1 KO had no obvious effect on localization of EEA1 and TGN46, markers of the early endosome and the TGN, respectively (Fig. 1). Notably, XXI treatment and PS1 KO increased the colocalization of GFP–DMT1-II and EEA1 compared to that seen in untreated control cells (Fig. 1A; Fig. S1) and decreased colocalization of GFP–DMT1-II and TGN46 (also known as TGOLN2) (Fig. 1B; Fig. S1), implying that γ-secretase is required for optimal retrograde trafficking of DMT1-II.

We performed rescue experiments to establish that the trafficking defect in PS1 KO cells was not due to an off-target effect. For these experiments, we used PS1 containing a FLAG epitope tag to allow us to use anti-FLAG antibody to identify cells expressing the introduced PS1 gene. HeLa control and PS1 KO cells were transfected with the plasmid expressing GFP–DMT1-II and co-transfected with the empty control plasmid or a plasmid expressing FLAG–PS1. As expected from the results presented above, in PS1 KO cells transfected with the control plasmid, GFP–DMT1-II accumulated in the EEA1 compartment at 24 hpi and was depleted from the TGN46 compartment compared to what was seen in control cells (Fig. 1C,D; Fig. S1). In contrast, trafficking of GFP–DMT1-II from the endosome to the TGN was largely restored in PS1 KO cells expressing FLAG–PS1. These results demonstrate that the defect of DMT1-II trafficking in the PS1 KO cells is due to loss of PS1 and not due to unintended off-target effects.

To explore whether γ-secretase plays a role in retromer-mediated trafficking of a second cellular protein, we examined CIMPR. As noted in the introduction, CIMPR can be transported by both retromer-dependent and retromer-independent pathways. Therefore, we first determined whether retromer KO had a detectable effect on trafficking of CIMPR in our cells. VPS35 and VPS26 double KO HeLa S3 (VPS35/26 KO) cells and HeLa S3 control cells were transduced with retrovirus expressing CD8–CIMPR, which consists of the extracellular domain of CD8 fused to the TM and cytoplasmic domain of CIMPR (Seaman, 2007). We assessed localization of CD8–CIMPR by immunofluorescence staining for CD8 and cell markers followed by confocal microscopy. Compared to control cells, colocalization of CIMPR with EEA1 was increased in VPS35/26 KO cells and colocalization of CIMPR with TGN46 was decreased (Fig. S2A,B), indicating that endosome-to-TGN trafficking of CIMPR is impaired in the retromer KO cells.

To assess whether γ-secretase facilitated CIMPR trafficking, PS1 KO HeLa cells, HeLa control cells, and control cells treated with XXI were transfected with a plasmid expressing CD8–CIMPR. γ-secretase inhibition or knockout increased CD8–CIMPR localization with EEA1 and decreased localization with TGN46 (Fig. S3A,B). In a rescue experiment, in PS1 KO cells transfected with the control plasmid, CD8–CIMPR accumulated in the EEA1 compartment at 24 hpi and was depleted from the TGN46 compartment, as expected (Fig. S3C,D). Notably, trafficking of CD8–CIMPR from the endosome to the TGN was largely restored in PS1 KO cells expressing FLAG–PS1 (Fig. S3C,D). Thus, as was the case for DMT1-II, the defect in retrograde trafficking of CIMPR in the PS1 KO cells is due to loss of PS1 and not due to unintended off-target effects.

The cytoplasmic domain of CIMPR present in CD8–CIMPR contains a tryptophan-leucine-methionine (WLM) retromer-binding site that mediates retromer-dependent trafficking of CIMPR from the endosome to the TGN. Replacing the WLM sequence with three alanine residues (AAA) inhibits retromer binding and retrograde trafficking (Seaman, 2007). We next determined whether γ-secretase affected trafficking of the CIMPR AAA mutant. HeLa control, XXI-treated control and PS1 KO cells were transfected with a plasmid expressing wild-type CD8–CIMPR or the CD8–CIMPR AAA mutant. As expected, the CD8–CIMPR AAA mutant accumulated in the endosome in untreated control cells (Fig. S2C), similar to what occurs in wild-type CD8–CIMPR in cells lacking γ-secretase. XXI and PS1 KO did not affect localization of the CD8–CIMPR AAA mutant. These results indicate that γ-secretase does not affect trafficking of CIMPR when this cargo cannot bind retromer and suggest that the inhibition of retrograde trafficking caused by γ-secretase inhibition is not the consequence of generalized cellular dysfunction.

### γ-secretase is required for efficient retromer-mediated retrograde trafficking of shiga toxin

The localization of cargoes that are expressed from transfected plasmids, as in the experiments described above, is determined by the balance of anterograde trafficking during synthesis or maturation and retrograde trafficking. To assess the effect of γ-secretase on retrograde trafficking in isolation, we studied transport of shiga toxin and cholera toxin, which depend on retrograde trafficking for cell entry (Bujny et al., 2007; Popoff et al., 2007; Sandvig and van Deurs, 2002). Importantly, these toxins can be added to cells as recombinant proteins and assayed acutely for localization without the confounding effects of new synthesis or anterograde transport.

**Fig. 2.**
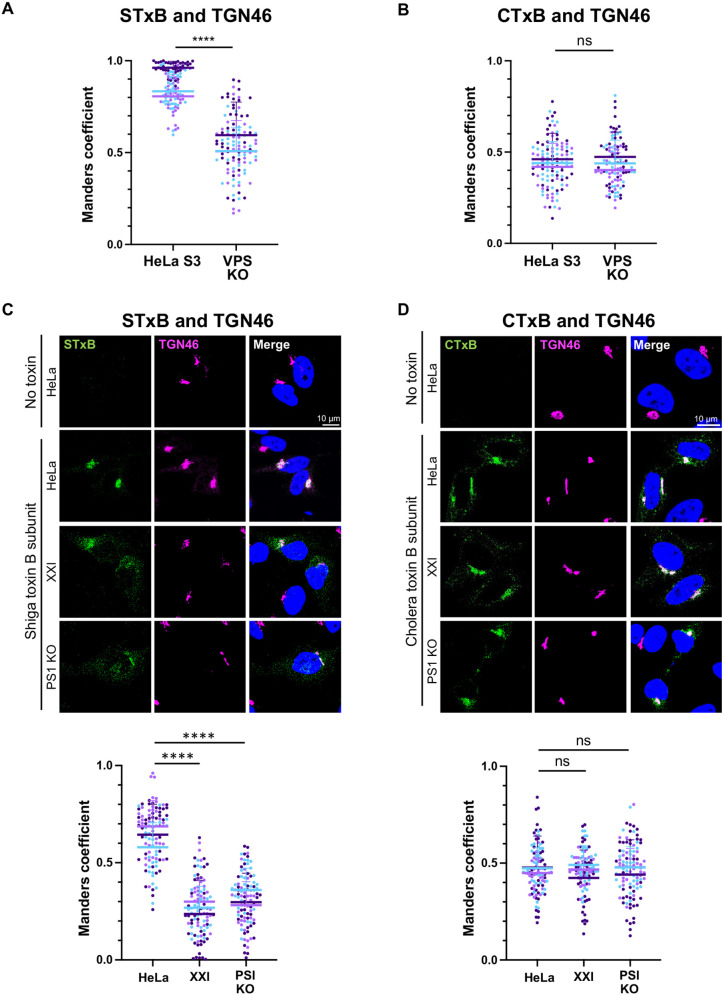
**γ-secretase inhibition and PS1 knockout inhibit retrograde trafficking of shiga toxin but not cholera toxin.** (A) HeLa S3 control cells (HeLa S3) and VPS35/26 KO HeLa S3 cells (VPS KO) were incubated with 1 μg/ml fluorescent Shiga toxin subunit B (STxB). Cells were fixed 30 min after the treatment and stained with DAPI and an antibody recognizing TGN46. Fluorescence confocal images were captured to determine overlap between STxB with TGN46 (representative images are in Fig. S3). Graph shows Manders coefficients for colocalization between STxB and TGN46 in cells containing detectable toxin. The graph and statistical analysis are as in Fig. 1A. ****P<0.0001 (two-tailed unpaired Student's *t*-test). (B) Same as A, except cells were incubated with 1 μg/ml fluorescent cholera toxin subunit B (CTxB). Graph shows Manders coefficients for colocalization between CTxB and TGN46 in cells containing detectable toxin. (C) HeLa control and PS1 KO HeLa cells were treated with DMSO or XXI for 24 h and then incubated with or without 1 μg/ml fluorescent STxB. Cells were fixed 30 min after treatment and stained with DAPI and an antibody recognizing TGN46. Fluorescence images of single confocal planes are shown: STxB, green; TGN46, magenta; nuclei, blue. Merged images show overlap between STxB and TGN46 pseudocolored white. Graph shows Manders coefficients for colocalization between STxB and TGN46 in cells containing detectable toxin. The graph and statistical analysis are as in Fig. 1A. (D) As in C, except cells were incubated with 1 μg/ml fluorescent (CTxB), and merged images show overlap between CTxB and TGN46. ****P<0.0001; ns, not significant (one-way ANOVA followed by a Tukey's multiple comparisons test)

First, we tested whether retromer was involved in trafficking of the transport subunits of shiga toxin and cholera toxin (STxB and CTxB, respectively) in our cells. We treated HeLa S3 control cells and VPS26/35 KO cells with fluorescent STxB or CTxB and assayed localization of these proteins 30 min later. As shown in Fig. 2A, Figs S1 and S4A, STxB showed tight, perinuclear localization in control cells, consistent with Golgi localization and displayed extensive overlap with TGN46. In the VPS26/35 KO cells, STxB displayed a more diffuse distribution and was markedly depleted in TGN46 compartments compared to control HeLa S3 cells. In contrast, the distribution of CTxB and its transport to the TGN was not affected by retromer knockout (Fig. 2B; Figs S1 and S4B). These results show that retromer is required for retrograde trafficking of STxB but not CTxB into the TGN46 compartment.

We next examined the acute effect of γ-secretase inhibition on trafficking of STxB and CTxB. HeLa control, XXI-treated control and PS1 KO cells were incubated with STxB for 30 min, and colocalization of STxB with TGN46 was analyzed by immunofluorescence. Compared to untreated control cells, XXI treatment and PS1 KO caused depletion of STxB from the TGN (Fig. 2C; Fig. S1), similar to the effect of retromer KO (Fig. 2A). Reduced transport of STxB to the TGN in the absence of γ-secretase function was confirmed when the TGN was stained with antibodies recognizing p230 (also known as GOLGA4), another TGN marker (Fig. S4C). We note that the TGN46 and p230 staining patterns are virtually identical under all conditions tested. These results indicate that γ-secretase is important for proper trafficking of STxB, a retromer-dependent retrograde cargo. In contrast, γ-secretase inhibition or knockout did not affect trafficking of CTxB, a retromer-independent cargo (Fig. 2D; Fig. S1). These data provide further evidence that γ-secretase inhibition impairs retrograde trafficking of retromer cargoes but does not cause a global disruption of retrograde trafficking.

XXI is known to inhibit the proteolytic activity of γ-secretase by binding PS1 (Seiffert et al., 2000). To confirm that the inhibition of trafficking by XXI was due to inhibition of γ-secretase activity, we treated HeLa control and PS1 KO cells with DMSO or XXI, incubated the cells with STxB for 30 min, and examined colocalization of STxB with TGN46 (Fig. S4D). As noted above, XXI treatment or PS1 knockout inhibited STxB trafficking. XXI did not cause further inhibition of STxB trafficking in the PS1 KO cells compared to the KO cells without XXI treatment. This result implies that the inhibition of trafficking by the γ-secretase inhibitor is not due to an off-target effect.

We performed a rescue experiment to confirm the role of γ-secretase in trafficking of STxB. PS1 KO cells were transfected with empty vector or with the vector expressing FLAG-tagged wild-type PS1 or the PS1 L166P mutant that lacks protease activity (Heilig et al., 2013; Koch et al., 2012). At 24 h after transfection, cells were treated with fluorescent STxB for 30 min, stained with antibodies recognizing TGN46 and FLAG, and examined by confocal microscopy. As expected, STxB displayed diffuse distribution and relatively low colocalization with TGN46 in the PS1 KO cells, consistent with impaired STxB trafficking, and wild-type PS1 rescued the trafficking defect. The catalytically inactive mutant did not mediate rescue (Fig. S5A), even though it was expressed at a similar level to wild-type PS1 (Fig. S5B). We note that, as previously reported, the distribution of the mutant PS1 is different from wild-type PS1 (Sannerud et al., 2016). The ability of wild-type but not mutant PS1 to support STxB trafficking provides additional evidence that γ-secretase is required for optimal trafficking of this cargo.

### γ-secretase and retromer do not affect the expression or localization of each other

**Fig. 3.**
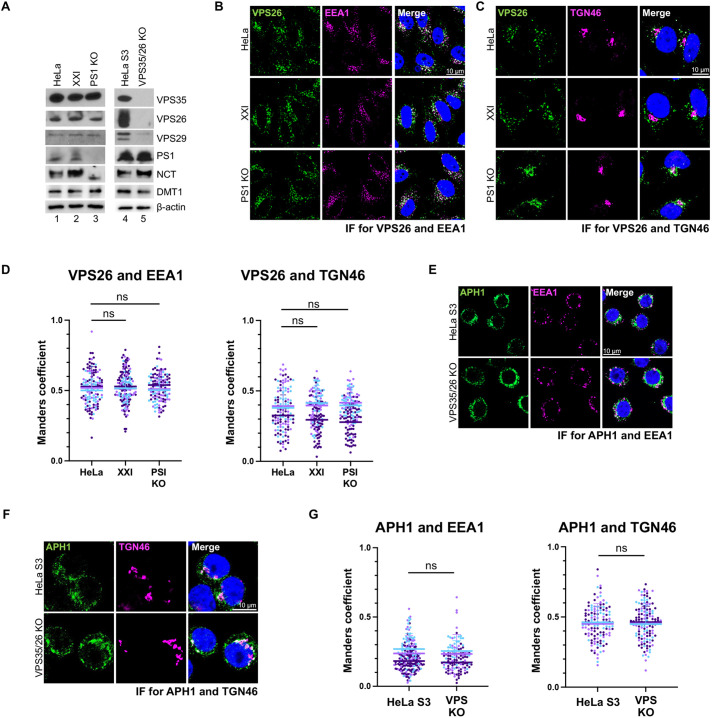
**Inhibition of γ-secretase does not affect expression or localization of the retromer complex and vice-versa.** (A) Detergent extracts were prepared from HeLa S3 control, VPS35/26 KO, HeLa control, and PS1 KO cells, and from HeLa control cells treated with 1 μM XXI for 24 h. Extracts were subjected to electrophoresis and western blot analysis using antibodies recognizing endogenous VPS35, VPS26, VPS29, PS1, NCT, DMT1-II and actin (as a loading control). Blot shown is representative of two repeats. (B) HeLa control and PS1 KO HeLa cells were treated with DMSO or 1 μM XXI for 24 h and then stained with DAPI and antibodies recognizing VPS26 and EEA1. Fluorescence images of single confocal planes are shown: VPS26, green; EEA1, magenta; nuclei, blue. Merged image shows overlap between VPS26 and EEA1 pseudocolored white. (C) As in B, except cells were stained with antibodies recognizing VPS26 and TGN46, and merged images shows overlap between VPS26 and TGN46. (D) Images as in B and C were quantified, and graphs shows Manders coefficients for colocalization of VPS26 and EEA1 (left panel) and VPS26 and TGN46 (right panel). ns, not significant (one-way ANOVA followed by a Tukey's multiple comparisons test). (E) HeLa S3 and VPS35/26 KO cells were stained with antibodies recognizing APH1 and EEA1, and merged image shows overlap between APH1 and EEA1 pseudocolored white. (F) Same as E, except cells were stained with antibodies recognizing APH1 and TGN46, and merged images shows overlap between APH1 and TGN46 pseudocolored white. (G) Images as in E and F were quantified, and graphs shows Manders coefficient for overlap between APH1 and EEA1 (left panel), and APH1 and TGN46 (right panel). The graphs and statistical analysis in D and G are as in Fig. 1A. ns, not significant (two-tailed unpaired Student's *t*-test).

We next determined whether γ-secretase affected expression and localization of retromer subunits and vice versa. We first performed western blotting of lysates of HeLa control, XXI-treated control and PS1 KO cells to confirm knockout of PS1 in the KO cells and show that XXI treatment did not affect the expression of endogenous PS1 (Fig. 3A, lanes 1–3). As expected, PS1 KO reduced expression of another γ-secretase subunit, nicastrin (NCT) (Fig. 3A, lane 3). Importantly, there was no difference in VPS35, VPS26 and VPS29 levels between HeLa control, XXI-treated control or PS1 KO cells (Fig. 3A, lanes 1–3). Similarly, KO of retromer subunits VPS35 and VPS26 reduced expression of VPS29 (as expected) but did not affect expression of the γ-secretase subunits PS1 and NCT compared to HeLa S3 control cells (Fig. 3A, lane 4 and 5). In addition, expression of endogenous DMT1-II was not changed by XXI treatment, PS1 KO, or VPS35/26 KO (Fig. 3A). These results show that absence of γ-secretase or retromer did not affect expression of each other or of DMT1-II.

We next used immunofluorescence to monitor the localization of VPS26 and APH1 in intact cells. Neither XXI treatment nor PS1 KO affected colocalization of VPS26 with EEA1 or TGN46 (Fig. 3B–D; Fig. S1). Similarly, VPS26/35 KO did not affect the colocalization of APH1 with EEA1 or TGN46 (Fig. 3E–G; Fig. S1). Thus, γ-secretase inhibition or KO had no apparent effect on the expression and localization of the retromer complex, and retromer KO did not affect the expression and localization of γ-secretase. In addition, inhibition of γ-secretase function had no discernable effect on the size or morphology of endosomes (Fig. 3B; Fig. S6A).

### Association of γ-secretase and retromer

**Fig. 4.**
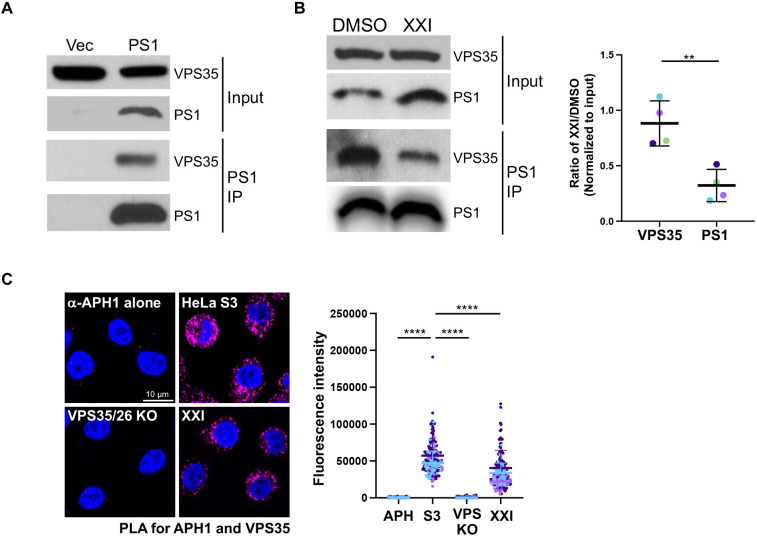
**γ-secretase and retromer interact in cell lysates and intact cells.** (A) PS1 KO cells were transfected with plasmids expressing VPS35, VPS26 and VPS29 and with empty vector or a plasmid expressing FLAG–PS1. At 24 hpt, detergent lysates were prepared, immunoprecipitated (IP) with an antibody recognizing PS1, and subjected to western blot analysis using an antibody recognizing VPS35 or PS1, as indicated. Blot of total lysates is shown as input. 2.25% and 47.75% of extract was used for input and IP, respectively. Similar results were obtained in two independent experiments. (B) Same as A, except HeLa cells expressing endogenous PS1 were transfected with plasmids expressing VPS35, VPS26 VPS29 and then treated with DMSO or 1 μM XXI. Graph shows mean±s.d. band intensity of protein expression in XXI-treated cells divided by expression in DMSO-treated cells, for four independent experiments. Each value is normalized to input. **P<0.01 (two-tailed unpaired Student's *t*-test). (C) HeLa S3 cells were treated with DMSO or 1 μM XXI for 24 h, and then PLA was performed with antibodies recognizing APH1 and VPS35. As negative controls, PLA was performed in VPS26/35 KO cells or in HeLa S3 cells with the VPS35 antibody omitted (α-APH1 alone). Images show single confocal planes. PLA signals are magenta; nuclei are stained blue with DAPI. The fluorescence of PLA signals was determined from multiple images (arbitrary units). Each dot represents PLA fluorescence intensity of an individual cell (with at least 30 cells counted per condition in each experiment); different colors are used to represent independent biological replicates. The statistical analysis is as in Fig. 1A (one-way ANOVA followed by a Tukey's multiple comparisons test).

A previous report used co-immunoprecipitation (co-IP) to show that PS1 interacts with VPS35 (Ueda et al., 2016). Here, we used co-IP to confirm the association of γ-secretase with retromer and determine whether γ-secretase inhibition affected the association. PS1 KO cells were transfected with plasmids expressing all three retromer subunits in addition to the empty control plasmid or a plasmid expressing FLAG–PS1. Cells were lysed in buffer containing the detergent CHAPSO and immunoprecipitated with an antibody recognizing PS1. The presence of VPS35 in the immunoprecipitates was determined by western blotting. Anti-PS1 co-immunoprecipitated (co-IPed) VPS35 from PS1-transfected PS1 KO cells, but not from PS1 KO cells lacking PS1 expression, indicating that PS1 interacts with VPS35 in our cells and co-IP was not due to cross-reaction of the PS1 antibody with VPS35 (Fig. 4A). Next, HeLa cells transfected to express the retromer subunits were treated with DMSO or XXI. Lysates were prepared and subjected to immunoprecipitation with antibody recognizing PS1. Consistent with the results shown in Fig. 4A, anti-PS1 co-IPed VPS35 from DMSO-treated cells. VPS35 was also co-IPed from XXI-treated cells, but XXI reduced the association of γ-secretase with retromer without inhibiting the expression of VPS35 or PS1 itself (Fig. 4B). Quantification of multiple independent repeats of this experiment showed that γ-secretase inhibition caused an ∼65% reduction in the amount of VPS35 co-IPed (Fig. 4B).

To explore whether γ-secretase and retromer interact in intact cells, we used a proximity ligation assay (PLA), which generates a fluorescent signal when two proteins of interest recognized by different antibodies are within 40 nm of one another. We performed PLA for APH1 and VPS35. As expected, only a low background level of PLA signal was detected in HeLa S3 control cells incubated with APH1 antibody alone and in VPS35/26 KO cells incubated with both VPS35 and APH1 antibodies (because these cells lack one of the PLA targets) (Fig. 4C). In contrast, clear PLA signals were observed when antibodies recognizing APH1 and VPS35 were used for PLA in HeLa S3 cells. Although PLA cannot demonstrate a direct interaction between the proteins of interest, these data strongly suggest that γ-secretase and retromer also interact in intact cells. In addition, XXI treatment reduced the APH1–VPS35 PLA signal (Fig. 4C; Fig. S1). The PLA signal between VPS35 and the catalytically inactive FLAG-tagged PS1 L166P was also reduced to a similar extent (Fig. S5C). Taken together, the co-IP and PLA results show that γ-secretase and retromer interact in cell lysates and in intact cells and that this interaction is inhibited but not abolished by inhibition of γ-secretase activity.

### γ-secretase is not required for association of retromer with cargo in intact cells

**Fig. 5.**
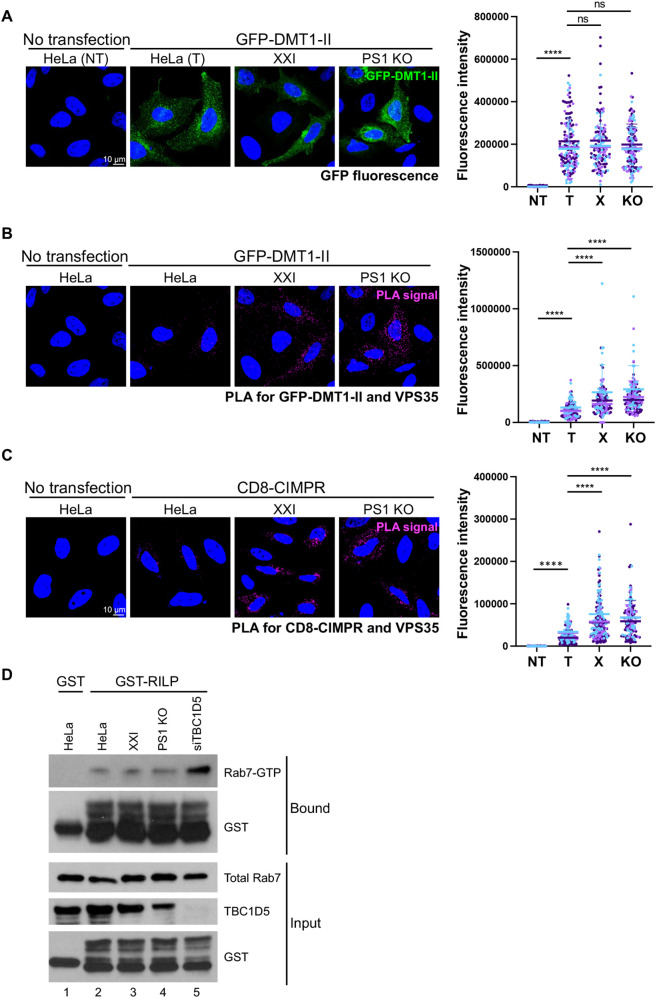
**γ-secretase inhibition and PS1 knockout do not block association of retromer and cargo nor increase the level of GTP-Rab7.** (A) HeLa control and PS1 KO HeLa cells were treated with DMSO or 1 μM XXI for 30 min and then mock transfected (NT) or transfected with a plasmid expressing GFP–DMT1-II. Cells were fixed 24 hpt and stained with DAPI. Single confocal planes are shown: GFP–DMT1-II, intrinsic green fluorescence; nuclei, blue. The GFP signals from multiple images was determined. Each dot represents GFP fluorescence intensity of an individual cell (with at least 30 cells counted per condition in each experiment); different colors are used to represent independent biological replicates. NT, not transfected; T, transfected control cells; X, transfected control cells treated with XXI; KO, PS1 KO cells. (B) Cells were treated as in A. After fixation, PLA was performed with antibodies recognizing GFP and VPS35. Single confocal planes are shown: PLA signals, magenta; nuclei, blue. These are the same fields of cells shown in A. PLA signals from multiple images was determined in cells expressing GFP–DMT1-II. (C) As in B, except cells were transfected with a plasmid expressing CD8-CIMPR, and PLA was performed with antibodies recognizing CD8 and VPS35. The statistical analysis for A–C is as in Fig. 1A; the intensity is in arbitrary units. ****P<0.0001; ns, not significant (one-way ANOVA followed by a Tukey's multiple comparisons test). (D) HeLa control cells were untransfected (lanes 1 and 2) and PS1 KO cells were transfected with siRNA targeting TBC1D5 (lane 5), treated with DMSO (lane 4), or treated with 1 μM XXI for 24 h (lane 3). At 48 hpt, detergent lysates were prepared and incubated with GST or GST–RILP, pulled down with glutathione agarose, and subjected to western blot analysis by using antibodies recognizing Rab7 or GST (panels labeled bound). Western blot of total lysates probed for Rab7, TBC1D5, and GST is shown as input (2%). Similar results were obtained in three independent experiments.

We next explored the basis for the defect in retrograde cargo trafficking caused by inhibition of γ-secretase activity and PS1 KO. Because γ-secretase inhibition did not affect the localization or expression of retromer (Fig. 3), we hypothesized that γ-secretase controls the association of retromer with cargo, so that inhibition or loss of γ-secretase impairs the ability of retromer to transport the cargo to the TGN from the endosome. We used PLA to test whether γ-secretase was required for the association of retromer and cargo. HeLa control, XXI-treated control and PS1 KO cells were mock transfected or transfected with a plasmid expressing GFP–DMT1-II. Expression of GFP–DMT1-II was not affected by XXI or PS1 KO (Fig. 5A; Fig. S1). PLA was then performed with antibodies recognizing GFP and VPS35. A low background level of PLA signal was detected in control cells not transfected with GFP–DMT1-II, and as expected a clear GFP–VPS35 PLA signal was detected in control cells expressing GFP–DMT1-II, indicating association of VPS35 and DMT1-II (Fig. 5B; Fig. S1). There was also abundant GFP–VPS35 PLA signal in XXI-treated and PS1 KO cells compared to that seen in control cells, indicating that γ-secretase was not required for the association of retromer and DMT1-II. Similarly, γ-secretase inhibition or KO did not decrease the CIMPR–VPS35 PLA signal (Fig. 5C; Fig. S1). In fact, γ-secretase inhibition or KO resulted in increased PLA signal between retromer and both cargo proteins. These data show that inhibition of γ-secretase activity and PS1 KO do not reduce the association of the cargo and retromer.

### γ-secretase inhibition does not affect levels of Rab7 or Rab7-GTP

To initiate retromer-mediated retrograde trafficking, the retromer complex and cargo are recruited to the endosome membrane under the control of the small GTPase Rab7. A Rab7 GAP, TBC1D5, stimulates the conversion of GTP-Rab7 to GDP-Rab7. Manipulations that increase the level of GTP-Rab7, e.g. expression of a constitutively active Rab7 mutant or knockdown of TBC1D5, increase association of cargo with retromer, whereas GTP hydrolysis promotes the release of retromer from cargo and endosomes (Seaman et al., 2018; Xie et al., 2020).

Because Rab7 controls the association and dissociation of cargo with retromer, we assessed the effect of γ-secretase inhibition on Rab7 levels. XXI treatment and PS1 KO did not change the abundance of total Rab7 compared to that seen in untreated HeLa control cells, as assessed by western blotting (Fig. 5D). To test whether γ-secretase inhibition increased the abundance of GTP-bound Rab7, which might account for the increased PLA signal between VPS35 and cargo in the inhibited cells, we performed pull-downs with recombinant Rab-interacting lysosomal protein (RILP), which binds GTP-bound but not GDP-bound Rab7. HeLa control, XXI-treated control, and PS1 KO cells were transfected with control siRNA and control cells were separately transfected with siRNA targeting the Rab7 GAP TBC1D5. Purified glutathione S-transferase (GST) or a GST–RILP fusion protein was incubated with lysates prepared from cells 48 h after siRNA transfection. Protein complexes were pulled down with glutathione beads and blotted with anti-Rab7 antibody to detect GTP-Rab7 (Fig. 5D). As expected, in lysates from untreated control cells, GTP-Rab7 was pulled-down with GST–RILP but not with the negative control reagent GST. Knockdown of TBC1D5 caused an increase in GTP-Rab7, also as expected (compare lane 5 to lane 2). However, neither XXI treatment nor PS1 KO changed the amount of Rab7 pulled down by GST–RILP (compare lane 3 and 4 to lane 2). These results indicate that loss of γ-secretase activity did not affect the level of GTP-Rab7. Thus, changes in the level of GTP-Rab7 are not responsible for the decrease in retromer-mediated trafficking or increased retromer-cargo association when γ-secretase is inhibited.

### γ-secretase associates with retromer cargo

**Fig. 6.**
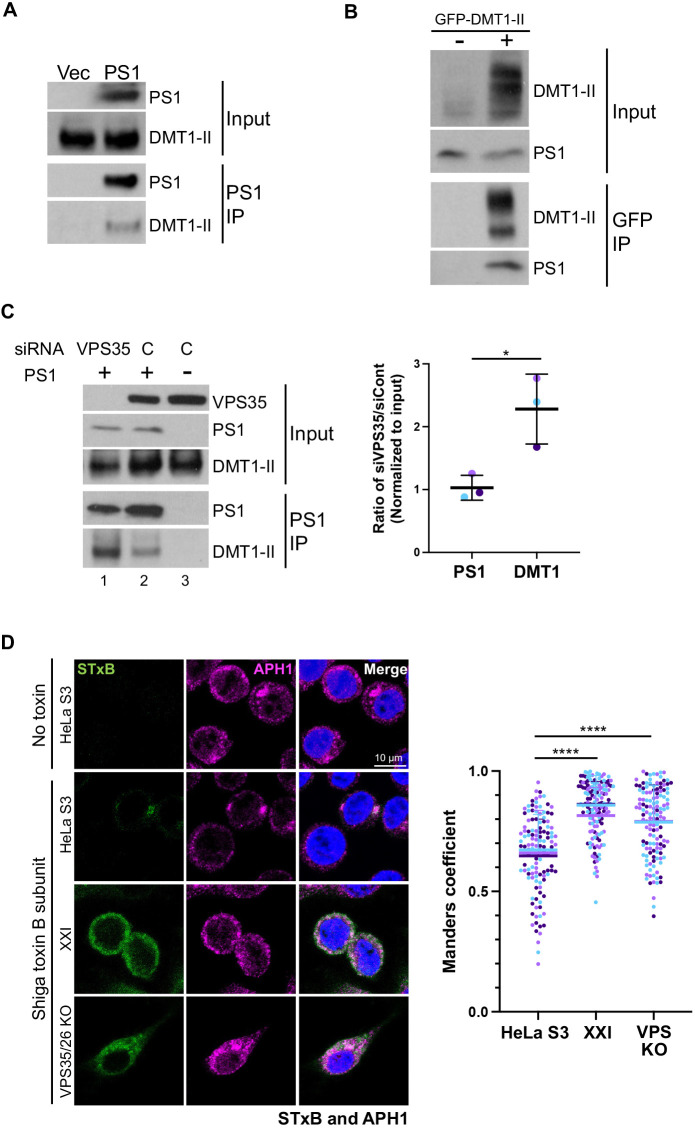
**γ-secretase associates with retromer cargo.** (A) PS1 KO cells were transfected with plasmids expressing all three retromer subunits and either empty vector or a plasmid expressing FLAG-tagged PS1. At 24 hpt, detergent extracts were prepared, immunoprecipitated with antibody recognizing PS1, and subjected to SDS-PAGE and western blotting for PS1 or endogenous DMT1-II (panels labeled PS1 IP). Samples in the absence of immunoprecipitation are shown as input. (B) 293T cells were transfected with plasmids expressing all three retromer subunits, FLAG-tagged PS1, and either a control plasmid or a plasmid expressing GFP–DMT1-II. At 24 hpt, detergent extracts were prepared and immunoprecipitated with GFP-Trap agarose beads. Samples were subjected to SDS-PAGE and western blotting with an antibody recognizing DMT1-II or PS1 (panels labeled GFP-IP). Sample not subjected to immunoprecipitation are labeled input. Blots shown in A and B representative of two repeats. (C) PS1 KO cells were transfected with siRNA targeting VPS35 (lane 1) or with control siRNA (denoted C, lane 2 and 3) for 24 h. Cells were then transfected with a plasmid expressing GFP-DMT1-II and either empty vector (lane 3) or a plasmid expressing FLAG-tagged PS1 (lane 1 and 2). At 24 hpt, detergent extracts were prepared, immunoprecipitated with antibody recognizing PS1, and subjected to SDS-PAGE and western blotting for PS1 or DMT1-II (panels labeled PS1 IP). Samples in the absence of immunoprecipitation are shown as input. Graph shows mean±s.d. band intensity of protein expression from siVPS35-treated cells (lane 1) divided by siControl-treated cells (lane 2), for three independent experiments. Each value is normalized to input. *P<0.05 (two-tailed unpaired Student's *t*-test). Inputs in A–C are 2%. (D) HeLa S3 and VPS26/35 KO cells were treated with DMSO or 1 μM XXI for 24 h and then incubated with 1 μg/ml fluorescent STxB for 30 min. Cells were fixed and stained with DAPI and an antibody recognizing APH1. Fluorescent confocal images were captured to determine overlap between STxB with APH1. Graph shows Manders coefficients for colocalization between STxB and APH1 in cells containing detectable toxin. The graph and statistical analysis are as in Fig. 1A. ****P<0.0001 (one-way ANOVA followed by a Tukey's multiple comparisons test).

Finally, we tested whether γ-secretase associates with cargo. First, we used co-IP to test whether γ-secretase and DMT1-II are in a physical complex. PS1 KO HeLa cells were transfected with plasmids expressing all three retromer subunits and either empty vector or a plasmid expressing FLAG-tagged PS1. At 24 hpt, detergent extracts were prepared, immunoprecipitated with antibody recognizing PS1, and subjected to SDS-PAGE and western blotting for endogenous DMT1-II. As shown in Fig. 6A, DMT1-II was co-IPed only from cells expressing PS1. Similarly, in a reciprocal co-IP experiment in extracts of 293T cells, PS1 was immunoprecipitated with a nanobody recognizing GFP from cells expressing GFP–DMT1-II but not from cells lacking GFP–DMT1-II expression (Fig. 6B). These results show that a complex between γ-secretase and the retromer cargo DMT1-II was detectable in cell extracts.

To determine whether retromer was required for association between γ-secretase and DMT1-II, we used siRNA to knockdown VPS35 and then assessed the ability of antibody recognizing PS1 to co-IP DMT1-II. As shown in Fig. 6C, lanes 2 and 3, DMT1-II was co-IPed from extracts of cells expressing PS1 but not from PS1 KO cells, as expected. DMT1-II was also co-IPed from cells depleted of retromer subunit VPS35 (lane 1), demonstrating that retromer was not required for the association between γ-secretase and its cargo, DMT1-II. In fact, the amount of DMT1-II in the co-immunoprecipitate was ∼2.2-fold more in the cells depleted of VPS35 (Fig. 6C).

To test whether γ-secretase and a different retromer cargo associate in intact cells, we used immunofluorescence to determine whether γ-secretase and STxB colocalize. HeLa S3 control, XXI-treated control and VPS35/26 KO cells were treated with fluorescent STxB for 30 min, and the localization of APH1 and STxB was analyzed by immunostaining and confocal microscopy. As shown in Fig. 6D and Fig. S1, STxB colocalized with APH1 in untreated control cells. In cells treated with XXI or lacking retromer, STxB displayed more diffuse distribution, as noted above in Fig. 2C and Fig. S4A, but these treatments did not disrupt the association between STxB and γ-secretase. In fact, colocalization of APH1 and STxB was increased in the retromer KO cells and the XXI-treated cells compared to untreated control cells, consistent with the increased co-IP of DMT1-II with PS1 in the cells depleted of VPS35 (Fig. 6C). These results show that γ-secretase and the retromer cargo STxB display overlapping localization in cells, which is increased by inhibition of γ-secretase activity or retromer KO.

We also note that there is considerable cell-to-cell heterogeneity in the pattern of APH1 staining, even within cell populations receiving the same treatment (Fig. 6D; Fig. S6B). There was no evident correlation between APH1 staining and the effect of various treatments on STxB trafficking. There are also slight differences in STxB localization in cells treated with XXI or depleted of retromer, which might reflect incomplete inhibition by XXI or compensatory changes in the KO cells during their derivation, although the inhibition of the arrival of STxB in the TGN in response to either treatment was clear.

## DISCUSSION

Retromer plays a key role in retrograde trafficking of certain intracellular TM proteins, whereas the protease γ-secretase cleaves TM protein targets. Retromer is reported to interact with γ-secretase in neuronal cells, and dysfunction of retromer or γ-secretase is implicated in the pathogenesis of neurodegenerative diseases (Kelleher and Shen, 2017; Reitz, 2018; Ueda et al., 2016; Vilariño-Güell et al., 2011; Wen et al., 2011), but prior work did not determine whether γ-secretase is required for retromer function. Here, we show that γ-secretase activity facilitates retromer-mediated retrograde trafficking of cellular TM proteins.

In this work, we used imaging studies to show that endosome-to-TGN trafficking of cellular TM protein retromer cargoes was impaired when γ-secretase was inhibited or knocked out. We observed impaired trafficking of two human proteins, DMT1-II and CIMPR, which were newly synthesized from transfected plasmids, and STxB, a recombinant bacterial toxin subunit that was acutely added to cells. In contrast, γ-secretase inhibition did not affect trafficking of a CIMPR mutant that is not recognized by retromer, nor did it affect trafficking of CTxB, a bacterial toxin whose trafficking is not dependent on retromer (Bujny et al., 2007; Seaman, 2007). Thus, in this study γ-secretase inhibition specifically inhibited retromer-mediated trafficking. Retrograde-mediated trafficking was not abolished in the PS1 KO cells, indicating that γ-secretase facilitates retromer-mediated trafficking but is not absolutely required for trafficking.

Two of the cargoes we studied use different SNX–retromer complexes. STxB requires the SNX-BAR protein SNX-1, whereas DMT1-II requires SNX-3, which lacks a BAR domain (Bujny et al., 2007; Lucas et al., 2016). The inhibition of both cargoes by γ-secretase inhibition implies that γ-secretase stimulates multiple types of retromer-mediated retrograde trafficking events. It is also possible that γ-secretase is involved in some non-retromer trafficking events. Further experiments are required to identify the specific trafficking pathways that are facilitated by γ-secretase.

It has been previously reported that γ-secretase interacts with retromer in detergent lysates of cultured murine neuroblastoma neuro2a cells and in homogenized mouse brain (Ueda et al., 2016). Here, we used co-IP to show that retromer and γ-secretase also associate in lysates of cultured human epithelial cells. Furthermore, our PLA studies imply that this interaction also occurs in intact cells. Thus, the association of retromer and γ-secretase occurs in a range of cell types and is not an artifact of cell lysis. The γ-secretase–retromer interaction was inhibited by XXI treatment or the L166P PS1 mutation, both of which decrease trafficking, suggesting that the association of γ-secretase with retromer is linked to its ability to support retrograde trafficking. Our co-IP and immunostaining data also showed that retromer cargo and γ-secretase associate or colocalize (Fig. 6). These results raise the possibility that optimal retrograde transport requires a ternary complex between retromer, cargo and γ-secretase.

We have ruled out several potential mechanisms by which γ-secretase inhibition might impair retromer-mediated retrograde trafficking. PS1 KO or inhibition did not appear to affect expression of any of the retromer subunits or localization of VPS26, suggesting that the inhibitory effect of impairing γ-secretase function is not due to altered expression or localization of retromer. Although retromer is required for proper expression and localization of PS1 in murine neuronal cells when the endocytic pathway is perturbed by treatment with agents such as NH_4_Cl that inhibit lysosomal function (Ueda et al., 2016), no effects on PS1 were observed in the absence of these treatments, consistent with our results. In addition, γ-secretase is not required for association of retromer with its cargo, implying that γ-secretase acts after retromer–cargo association.

We also showed that γ-secretase and the retromer cargoes DMT1-II and STxB interact, even in cells lacking retromer. Thus, γ-secretase and retromer can each associate with cargo in the absence of the other factor. In fact, γ-secretase inhibition or KO was accompanied by increased retromer–cargo association, and γ-secretase inhibition and retromer KO increased γ-secretase–cargo association. Rab7-GTP normally recruits cargo to retromer, but the increased retromer–cargo association upon γ-secretase inhibition is not due to elevated Rab7-GTP. It is possible that reduced dissociation of cargo from retromer in response to γ-secretase inhibition contributes to impaired trafficking, but expression of constitutively active Rab7 decreases CIMPR–retromer dissociation without inhibiting trafficking (Jimenez-Orgaz et al., 2018; Xie et al., 2020). Therefore, the increased retromer–cargo association is likely to be a consequence of inhibition of trafficking, not the cause of it.

The mechanistic role of γ-secretase in retrograde trafficking remains to be determined. Retrograde transport of cargo to the TGN is a complex process that requires many proteins acting after retromer interacts with cargo. For example, other proteins contribute to tubule and vesicle formation and movement to the TGN, including sorting nexins and the WASH complex, which regulates actin cytoskeletal dynamics during retromer function (Johannes and Popoff, 2008). γ-secretase action might be required for optimal recruitment or function of these factors. It is possible that γ-secretase cleaves a protein required for efficient trafficking. Consistent with this model, the chemical γ-secretase inhibitor XXI and the L166P PS1 mutation, which abolishes catalytic activity, inhibit γ-secretase cleavage activity and interfere with trafficking (Koch et al., 2012). However, both of these manipulations also inhibit the interaction between γ-secretase and retromer, and the L166P mutation affects the intracellular distribution of PS1 (Fig. S5A) (Sannerud et al., 2016). Therefore, it is not clear that proteolytic activity of γ-secretase per se is required for trafficking. In addition, retromer subunits, SNX proteins, and Rab7 all lack TMDs, the substrate for γ-secretase cleavage. Furthermore, γ-secretase does not typically cleave the TMD of the cargo itself because retrograde cargo cleavage is not commonly observed during retromer-mediated trafficking, and in our experiments, inhibiting γ-secretase did not affect the level of full-length DMT1-II (Fig. 3A). Thus, if the proteolytic activity of γ-secretase is involved in retrograde trafficking, it presumably acts indirectly by cleaving protein(s) other than retromer, its cargo or sorting nexins. Alternatively, γ-secretase might play a non-proteolytic role in trafficking, such as serving as a scaffold to assemble a protein complex important for trafficking. The presence of TMDs in both the retromer cargoes and γ-secretase itself raises the possibility that any such a scaffolding function may involve interactions between TMDs.

Our finding that retromer and γ-secretase are both required for efficient retrograde trafficking may have relevance to neurodegenerative diseases. Mutations in PS1 and other genes encoding γ-secretase subunits can cause familial AD and PD (Forloni et al., 2002; Kelleher and Shen, 2017). γ-secretase catalyzes intramembranous proteolysis of amyloid precursor protein (APP) and generates Aβ, the accumulation of which is thought to be key in the pathogenesis of AD (Hur, 2022). The biochemical role of γ-secretase in PD is unclear, but there is a report that istradefylline, an anti-PD drug, enhances Aβ generation and γ-secretase activity (Lu et al., 2016). It is also unclear how retromer contributes to the pathogenesis these diseases, although perturbations in endocytic trafficking have been implicated in AD (Kanatsu et al., 2018; Reitz, 2018; Vagnozzi and Praticò, 2019; Zhang et al., 2018a). Mutations in retromer subunits, including VPS35 D620N, can cause PD, and retromer levels are reduced in the brains of individuals with late-onset AD (Muhammad et al., 2008). The VPS35 D620N mutation increases colocalization of CIMPR with VPS35 and impairs trafficking of CIMPR and other retromer cargoes (McGough et al., 2014), similar to the pattern we observed when γ-secretase is inhibited. We speculate that some γ-secretase mutations might inhibit retrograde trafficking of a variety of retromer cargoes that contribute to disease pathogenesis. For example, SORL1 is a retromer cargo, and mutations in SORL1, which affects APP recycling, can cause familial AD (Mishra et al., 2022). Thus, impairment of retromer activity through mutations in γ-secretase might affect SORL1 function. It will be interesting to determine whether some γ-secretase mutations associated with neurodegenerative disease contribute to pathogenesis by affecting retromer-mediated retrograde trafficking.

In summary, we show that γ-secretase is required for optimal retromer-mediated retrograde trafficking of cellular proteins from the endosomes to the TGN. As well as providing new insight into the regulation of intracellular protein trafficking, the activities reported here might also have implications for the pathogenesis and possibly treatment of devastating human disease.

## MATERIALS AND METHODS

Inhibitors and antibodies used in this study are listed in Tables S1–S3.

### Cell culture

HeLa S3 (CCL-2.2), HeLa (CCL-2), and HEK293T/17 (CRL-11268) cells were obtained from American Type Culture Collection (ATCC). All cell lines were cultured at 37°C and 5% CO_2_ in Dulbecco's modified Eagle's medium (DMEM; Sigma) supplemented with 10% fetal bovine serum (FBS; R&D Systems), L-glutamine (Gibco), 100 units/ml penicillin-streptomycin (Gibco) and 20 mM HEPES (Sigma) (DMEM10). Cell lines were verified by using the ATCC cell authentication service.

### Plasmid construction

Plasmids expressing 3×Flag–PS1 or 3×Flag–L166P PS1were constructed as follows. To generate the pCMVTNT-3xFlag-linker vector for subcloning, 3×Flag and a linker that contained BamHI and EcoRI sites were introduced between XhoI and BamHI sites of pCMVTNT-HA-L2-3xFLAG, a gift from Billy Tsai (University of Michigan, USA) using In-Fusion cloning (TaKaRa Bio, 638948). During In-Fusion cloning, we deleted the original BamHI site. The PS1 or L166P PS1 gene was amplified from pCAG-PS1 or pCAG-L166P PS1 (gifts from Billy Tsai), respectively, using primers 5′-GTGGTTCTGGTGGTGGATCCGGTACAGAGTTACCTGCACCGTTGTCCTACTTC-3′ and 5′-GCTCGAAGCGGAATTCCTAGATATAAAATTGATGGAATGCTAATTG-3′. The amplified segment was introduced between BamHI and EcoRI sites of pCMVTNT-3xFlag-linker by In-Fusion cloning. Resulting plasmids were confirmed by DNA sequencing. Plasmids are available upon request.

### Generation of KO cells and matched control cells

To generate VPS35 KO cells, oligonucleotides 5′-CACCGGGAGCATTTGCGCTTGCGGC-3′ and 5′-AAACGCCGCAAGCGCAAATGCTCCC-3′ comprising the VPS35 guide RNA were annealed and inserted into the pLentiCRISPRv2 (Addgene #49535) vector. pLentiCRISPRv2 plasmid expressing the VPS35 guide RNA was transfected into 293T cells using Lipofectamine 2000 with pMG2.D and psPAX2 packaging plasmids (Addgene #12259 and #12260). At 48 hpt, the growth medium containing lentivirus was harvested and filtered through a 0.45 μm filter. HeLa S3 cells were transduced with the lentivirus by using 4 μg/ml polybrene. The culture medium was replaced with fresh DMEM10 at 24 h post infection (hpi), and 0.4 μg/ml puromycin was added at 48 hpi. After selection, surviving HeLa S3 cells were diluted and seeded into 96-well plates, so that each well contained no more than a single cell. Wells were monitored for single colony growth. After cell expansion, knockout of VPS35 was confirmed with western blotting. In parallel, HeLa S3 cells were transduced with lentivirus lacking an sgRNA to generate HeLa S3 control cells. To generate VPS35/26 double KO cells, oligonucleotides 5′-CACCGTTCATACCTCAAGCGGACAT-3′ and 5′-AAACATGTCCGCTTGAGGTATGAAC-3′ comprising the VPS26 guide RNA were annealed and introduced into pLentiCRISPRv2 hygro (Addgene #98291). Lentivirus was produced as described above and transduced into VPS35 KO cells. Cells were selected with 0.25 mg/ml of hygromycin B (Thermo Fisher Scientific, 10687010), cloned, and tested by western blotting for VPS26 KO.

PS1 KO cells and HeLa control cells were gifts from Billy Tsai and Takamasa Inoue (University of Michigan, USA). PS1 KO cells were generated from HeLa (ATCC, CCL-2) cells as previously described (Inoue et al., 2018). To generate HeLa control cells for experiments involving the PS1 KO cells, oligonucleotides 5′-CACCGCGGGAGATCCTTGGGGCGGT-3′ and 5′-TTTGACCGCCCCAAGGATCTCCCGC-3′ comprising the guide RNA of AAVS1 were annealed and introduced into pX330 vector (Addgene #110403), which was then transfected into HeLa (ATCC, CCL-2) cells.

### Immunofluorescence

2×10^5^ HeLa control and PS1 KO cells or 4×10^5^ HeLa S3 control and VPS35/26 KO cells were plated in 24-well plates containing glass coverslips 20 h prior to transfection. Cells were treated with DMSO or 1 μM XXI 30 min before transfection with 1 μg plasmid expressing protein of interest. At 24 hpt, cells were fixed with 4% paraformaldehyde (Electron Microscopy Sciences) at room temperature (RT) for 10 min and treated with 0.1% saponin (Sigma-Aldrich, 47036) in DMEM10 for 10–30 min at RT. Primary antibodies were diluted in 0.1% saponin in DMEM10 and incubated with the cells overnight at 4°C. Alexa-Fluor-conjugated secondary antibodies were also diluted in 0.1% saponin in DMEM10 and incubated with the cells at RT for 1 h. When the primary antibody recognizing VPS26 or APH1 was used, we used Alexa Fluor 647-conjugated anti-TGN46 antibody that was prepared using Alexa Fluor Antibody Labeling Kits (Thermo Fisher Scientific, A20186) according to the manufacturer's instructions. Coverslips were mounted with mounting medium containing DAPI (Abcam, ab104139). Cellular fluorescence was imaged using the Zeiss LSM980 or LSM800 confocal microscope. Images were processed using a Zeiss Zen software version 3.1 and quantified using Image J software. Manders' coefficients were used to quantify the degree of colocalization between two channels and were calculated by using ImageJ software. Regions of interest from cells expressing the indicated cargo were masked manually. Data were analyzed from multiple images (typically >30 cells) for each experimental condition. Full details of antibodies used in this study are listed in Tables S2 and S3.

### Measurement of endosome size

Endosome size was quantified from EEA1-stained images using Image J software. Regions of interest (ROIs) from the experiment shown in Fig. 3B were drawn around EEA1 puncta with a minimum size of 0.106 μm^2^ (3 pixels). The endosome size was measured and reported as area (μm^2^). Data were analyzed from multiple random cells per image of the respective sample.

### GFP–DMT1-II and CD8–CIMPR trafficking assays

Cells were plated as above and treated with DMSO or 1 μM XXI for 30 min. 1 μg of pGFP-DMT1-II (obtained from Mitsuaki Tabuchi, Kagawa University, Japan) or CD8-CIMPR plasmid (obtained from Matthew Seaman, Cambridge Institute for Medical Research, UK) was transfected into the cells by using Trans-IT HeLaMONSTER reagent (Mirus Bio). DMSO and XXI were maintained in the culture medium during transfection. At 24 hpt, cells were fixed and processed for immunofluorescence as described above.

### Toxin uptake assays

Cells plated as described above were treated with DMSO or 1 μM XXI for 24 h and then incubated for 30 min with 1 μg/ml of Cholera Toxin Subunit B, Alexa Fluor 488 Conjugate (Thermo Fisher Scientific, C34775) or Shiga toxin 1 B subunit (Sigma, SML0562), conjugated with Alexa Fluor 555 using the Alexa Fluor 555 Microscale Protein Labeling Kit (Thermo Fisher Scientific, A30007) according to the manufacturer's instructions. DMSO and XXI were maintained in the culture medium during toxin incubation. Cells were fixed 30 min after toxin treatment and processed for immunofluorescence as described above.

### Western blot analysis

Cells were lysed using ice-cold radioimmunoprecipitation assay (RIPA; 50 mM Tris-HCl pH 7.4, 150 mM NaCl, 1% Nonidet P-40, 1% sodium deoxycholate, 0.1% SDS, 1 mM EDTA) buffer supplemented with 1× HALT protease and phosphatase inhibitor cocktail (Pierce Thermo Fisher Scientific) for 15 min at 4°C. After centrifugation at 16,000 ***g*** for 15 min in an Eppendorf 5430R centrifuge, the supernatant was mixed with 4× Laemmli dye (Bio-Rad) supplemented with 10% 2-mercaptoethanol and incubated for 5 min at 100°C. Samples were then separated by SDS-PAGE (4–12% acrylamide; Bio-Rad) and analyzed by western blotting. Secondary horseradish peroxidase (HRP)-conjugated antisera recognizing rabbit or mouse antibodies as appropriate (Jackson ImmunoResearch, 711-035-152, 115-035-146) were used at 1:5000 to 1:10,000 dilution. The blots were developed with SuperSignal West Pico or Femto Chemiluminescent substrate (Pierce) and visualized by using FluorChem Imager (Bio-technne, FE0685) or film processor (Fujifilm). Uncropped images of western blots from this study are shown in Fig. S7.

### Co-immunoprecipitation

HeLa control cells, PS1 KO HeLa, or 293T cells were plated in 60 mm or 10 cm dishes. Polyethylenimine (PEI) was used to transfect PS1 KO cells with three plasmids (3 μg each) encoding retromer subunits (VPS35, VPS26, and VPS29), originally constructed by Carol Haft (NIDDK), were obtained from Jae Jung (University of Southern California, USA) and Nam-Hyuk Cho (Seoul National University, Republic of Korea) (Kingston et al., 2011), and 3 μg of a control plasmid or a plasmid expressing FLAG-tagged PS1, constructed as described above. HeLa control cells were transfected with the plasmids encoding retromer subunits and treated with DMSO or 1 μM XXI for 24 h. 293T cells were transfected with the plasmids encoding retromer subunits, FLAG-tagged PS1 and a control plasmid or a plasmid expressing GFP–DMT1-II. 7×10^6^ PS1 KO HeLa cells were plated in 10 cm dishes and transfected with 10 nM of control or VPS35 siRNA (Dharmacon, D-001810-10-05 and L-010894-00-0005, respectively) using Lipofectamine RNAiMAX Transfection Reagent (Thermo Fisher Scientific, 13778100) in the presence of DMSO or 1 μM XXI. After 24 h, PEI was used to transfect the cells with 4 μg of a plasmid expressing GFP–DMT1-II and 4 μg of a control plasmid or a plasmid expressing FLAG-tagged PS1. After 24 h, cells were washed with ice-cold Dulbecco's phosphate-buffered saline (DPBS) and lysed in 500 μl of lysis buffer [50 mM HEPES pH 7.4, 150 mM NaCl, 1% CHAPSO (Sigma, C3649)] supplemented with 1× HALT protease and phosphatase inhibitor cocktail. The lysate was centrifuged as above, and the supernatant was transferred to new tubes. 10% of supernatant was reserved for input samples. The remainder of the supernatant was incubated with 25 μl of GFP-trap agarose beads (chromotek) or 1 μl of anti-PS1 antibody for 3 h or overnight at 4°C, then incubated with 20 μl of protein G magnetic beads (Thermo Fisher Scientific) for 1 h at 4°C. Bound proteins were collected with a magnet, washed four times with lysis buffer, and eluted with 40 μl of 2x Laemmli sample buffer (Bio-Rad) containing 5% 2-mercaptoethanol for 5 min at 100°C, followed by SDS-PAGE and western blot analysis.

### Western blot band intensity analysis

Band intensities of western blot images were quantified using Image J Fiji (version 2.14.0/1.54f) software. Bands were identified, and their intensity were measured. Background noise was subtracted based on the same size of area of the blot lacking bands. Normalization was performed by using the intensity of the input sample.

### Proximity ligation assay

2×10^5^ HeLa control and PS1 KO cells or 4×10^5^ HeLa S3 control and VPS35/26 KO cells were plated per well in 24-well plates containing glass coverslips. 1 μg of pGFP-DMT1-II or CD8-CIMPR plasmid was transfected into the cells by using Trans-IT HeLaMONSTER reagent or treated with DMSO or 1 μM XXI for 24 h. Cells were fixed, permeabilized and blocked as described above. Cells were then incubated overnight at 4°C with a pair of mouse and rabbit antibodies recognizing the proteins of interest. PLA was performed with Duolink reagents (Sigma) according to the manufacturer's instructions. Briefly, cells were incubated in a humidified chamber at 37°C with pairs of PLA antibody probes (mouse and rabbit) for 1 h, with ligation mixture for 45 min, and then with amplification mixture for 3 h, followed by series of washes. Coverslips were mounted with mounting medium containing DAPI. Cellular fluorescence was imaged using the Zeiss LSM980 or LSM800 confocal microscope. Images were processed using a Zeiss Zen software version 3.1 and quantified by using Image J software to determine fluorescence intensity of PLA signals per cell.

### RILP pulldown assay

Plasmids expressing GST-RILP (obtained from Christopher Burd, Yale University, USA) in pGEX-KG vector (Addgene #77103) or pGEX KG expressing GST alone was transformed into *E.coli* strain BL21 (DE3). Bacterial cultures at an optical density at 600 nm (OD_600_) of 0.6 were induced with 0.4 mM isopropyl β-D-1-thiogalactopyranoside (IPTG) at 30°C for 6 h. Bacteria were harvested and lysed with B-per lysis buffer (Thermo Fisher Scientific) supplemented with 1× HALT protease inhibitor cocktail. GST–RILP or GST proteins were purified by using a pre-equilibrated slurry of glutathione agarose (Thermo Fisher Scientific, 16100) in Buffer I [50 mM Tris pH8.0, 150 mM NaCl, 1 mM MgCl_2_] and washed three times in the same buffer. Purified proteins were eluted from the beads with reduced 20 mM glutathione in Buffer I and dialyzed into HEPES buffer [20 mM HEPES pH7.4, 50 mM NaCl, 5 mM MgCl_2_, 1 mM dithiothreitol (DTT)] using Slide-A-Lyzer dialysis Cassette (Thermo Fisher Scientific). Protein amounts were quantified with bovine serum albumin (BSA) standards separated by SDS-PAGE followed by Coomassie Blue staining.

3×10^6^ HeLa control or PS1 KO HeLa cells were plated per dish in 60 mm dishes and transfected with 10 nM of control or TBC1D5 siRNA (Dharmacon, D-001810-10-05, L-020775-01-0005, respectively) using Lipofectamine RNAiMAX Transfection Reagent (Thermo Fisher Scientific, 13778100). Cells were then treated with DMSO or 1 μM XXI for 24 h. At 48 hpt, cells were washed with ice-cold DPBS and lysed using 300 μl RILP lysis buffer (HEPES buffer containing 0.15% Triton X-100) supplemented with 1× HALT protease and phosphatase inhibitor cocktail. After centrifugation at 16,000 ***g*** for 20 min at 4°C in an Eppendorf 5430R centrifuge, the supernatant was incubated with 10 μg of purified GST or GST-RILP proteins at 4°C for 2 h. After addition of GST proteins, 30 μl of mixtures were taken for input samples. Then 40 μl of pre-equilibrated slurry of glutathione agarose was added and the mixtures were further incubated at 4°C for 3 h. followed by three washes in lysis buffer. Bound proteins were eluted with 40 μl of 2× Laemmli sample buffer containing 5% 2-mercaptoethanol for 5 min at 100°C. Input and eluted samples were separated by SDS-PAGE, and western blot analysis was carried out as described above.

### Statistical analysis

Statistical analysis was conducted using GraphPad Prism (Version 10.0.3). For comparisons of two groups, unpaired Student's *t*-tests were applied. For comparisons of more than two groups, one-way ANOVA with the ordinary ANOVA test were applied. These analyses provide *P-*values for each comparison.

## Supplementary Material

10.1242/joces.263538_sup1 Supplementary information
