# Supplementary material for: γ-secretase facilitates retromer-mediated retrograde transport
Source: J Cell Sci. 2025 Feb 20;138(4):JCS263538. doi: 10.1242/jcs.263538 (PMC11883284; doi:10.1242/jcs.263538)
Supplement: Supplementary information [file joces-138-263538-s1.pdf]

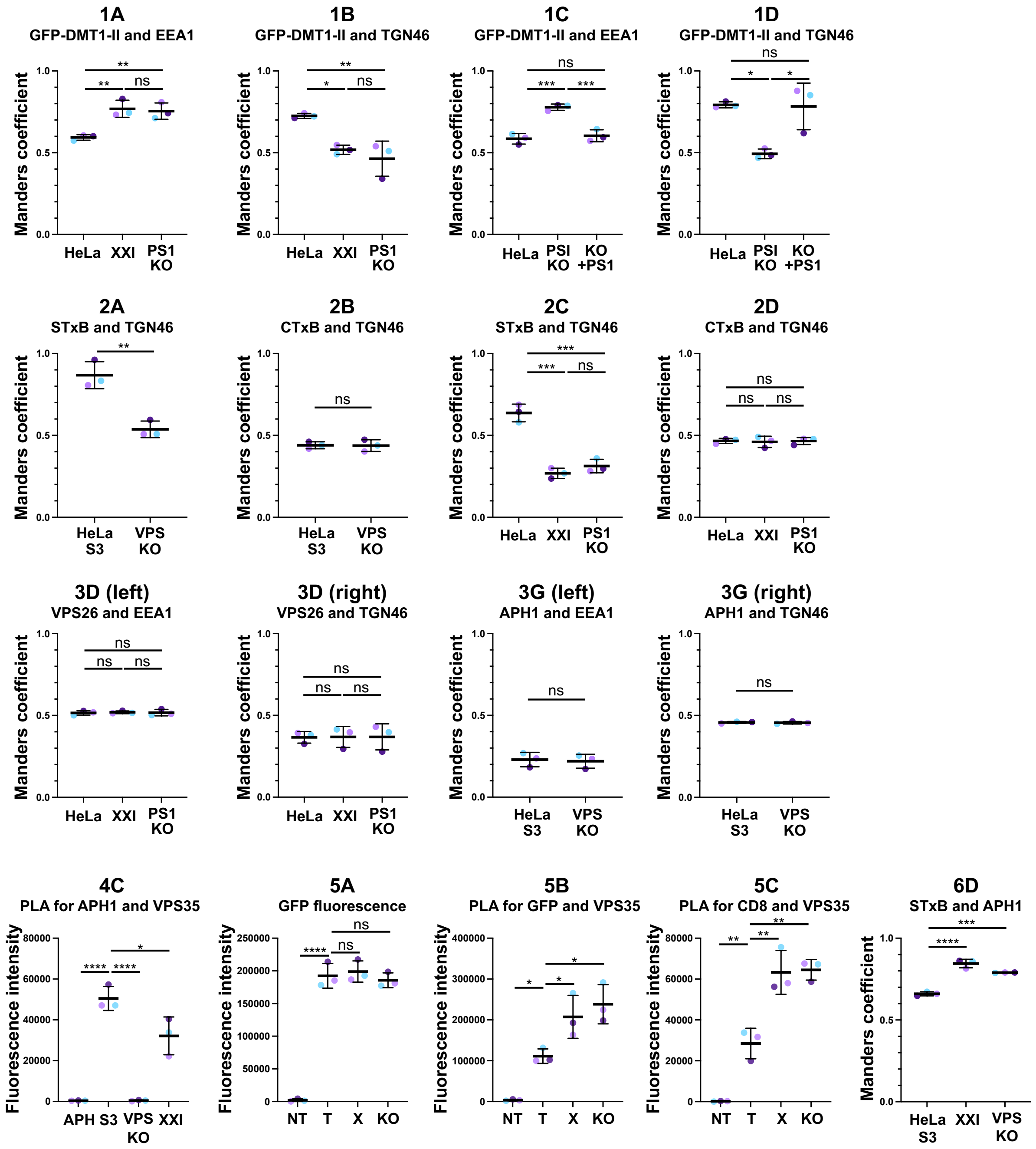

Fig. S1. Statistical analysis of independent biological replicates.

Each graph shows statistical analysis of the mean value of three biological replicates from main figures as indicated above each graph. For comparisons of two groups, unpaired *t*-tests were applied. For comparisons of more than two groups, One-way ANOVA with the ordinary ANOVA test were applied. *, $p<0.05$; **, $p<0.01$; ***, $p<0.001$; ****, $p<0.0001$; ns, not significant.

Journal of Cell Science • Supplementary information

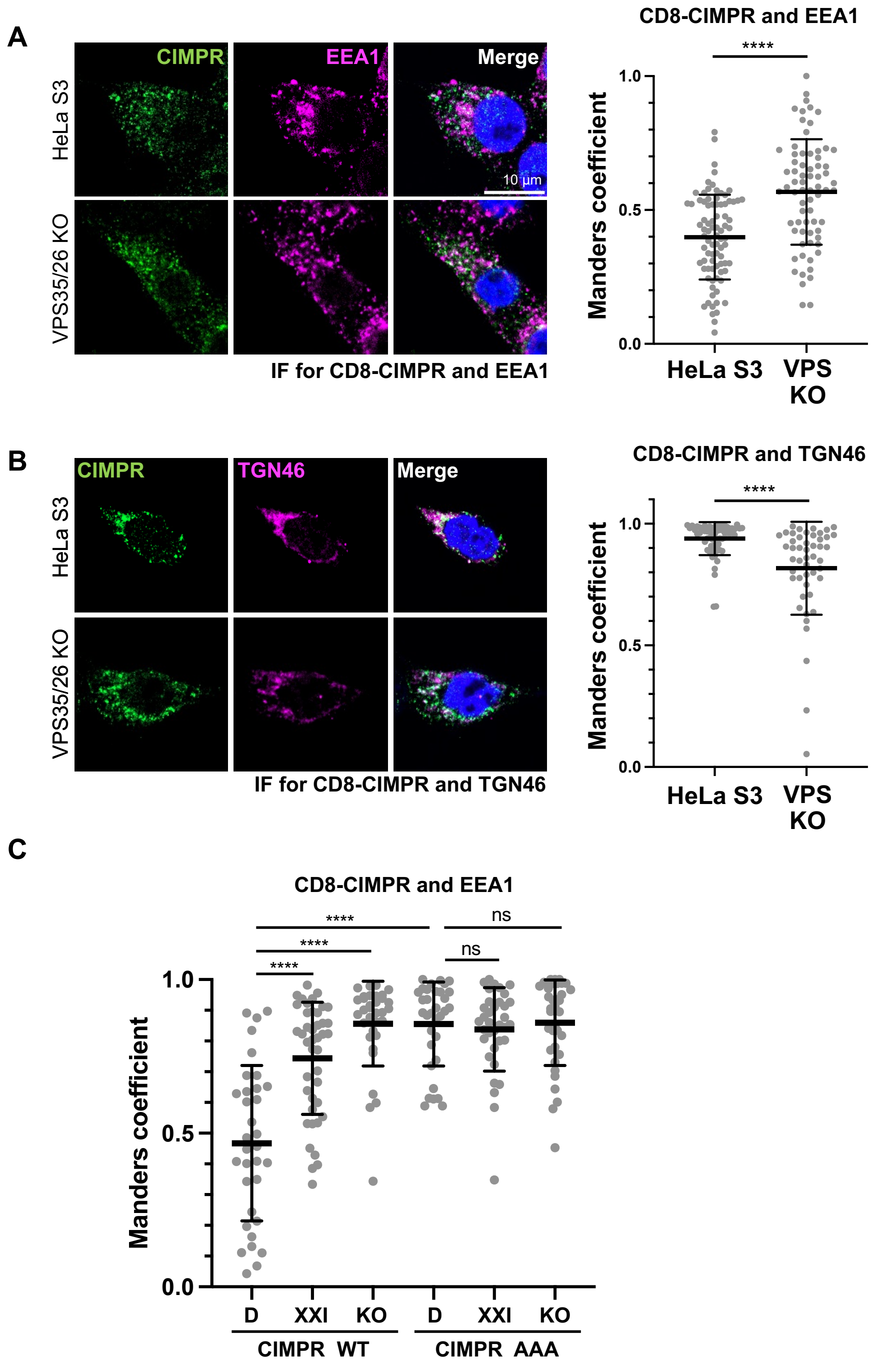

Fig. S2. Effect of retromer and γ-secretase on trafficking of wild-type and mutant CIMPR. (**A**) HeLa S3 control cells (HeLa S3) and VPS35/26 KO HeLa S3 cells (VPS KO) were infected with retrovirus expressing CD8-CIMPR. Cells were fixed 24 h post infection and stained with DAPI and antibodies recognizing CD8 and EEA1. Fluorescent images of single

Journal of Cell Science • Supplementary information

confocal planes are shown: CD8-CIMPR, green; EEA1, magenta; nuclei, blue. Merged images show overlap between CD8-CIMPR and EEA1 pseudocolored white. Graph shows Manders coefficients for colocalization of CD8-CIMPR and EEA1 in cells expressing CD8-CIMPR for a representative experiment. Each dot represents an individual cell (n>50) for a representative experiment, and horizonal line indicates the mean value of the analyzed population in each group. ****, $p<0.0001$. Similar results were obtained in two independent experiments. (**B**) As in (**A**) except cells were stained with antibodies recognizing CD8 and TGN46. Merged images show overlap between CD8-CIMPR and TGN46. (**C**) DMSO-treated HeLa control cells (D), XXI-treated control cells (XXI), and DMSO-treated PS1 KO HeLa cells (KO) were transfected with a plasmid expressing wild-type CD8-CIMPR (WT) or a AAA mutant CD8-CIMPR (AAA). Cells were fixed 24 hpt and stained with antibodies recognizing CD8 and EEA1. Graph shows Manders coefficients for CD8-CIMPR and EEA1 colocalization as described in (**A**) for a representative experiment. The graph and statistical analysis are as in Fig. S2A. ****, $p<0.0001$; ns, not significant. Similar results were obtained in two independent experiments.

Journal of Cell Science • Supplementary information

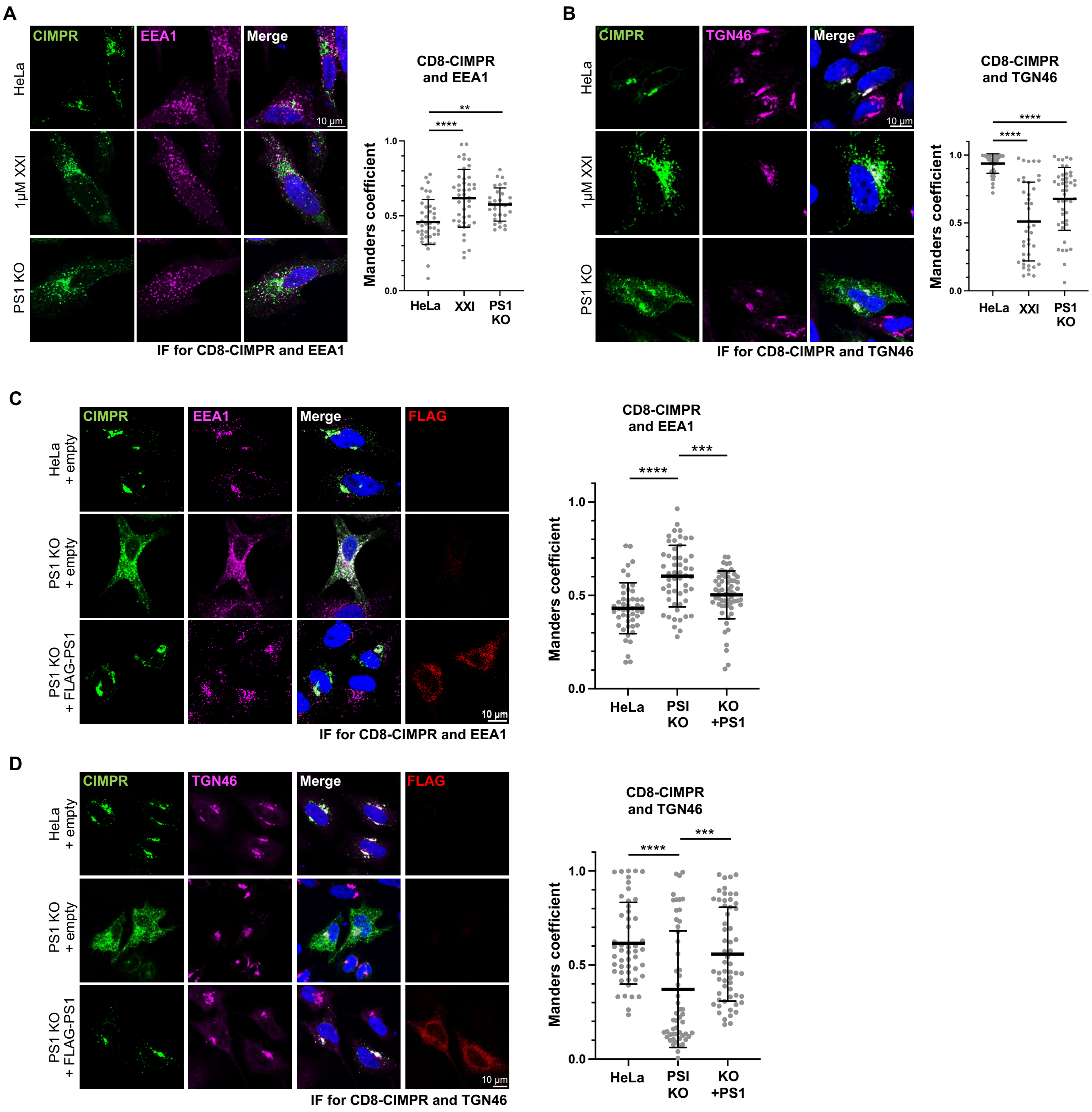

Fig. S3. γ-secretase inhibition and PS1 knockout inhibit retrograde trafficking of CIMPR. (**A**) HeLa control cells (HeLa) and PS1 knockout HeLa cells (PS1 KO) were treated with DMSO or 1 μM XXI for 30 min and then transfected with a plasmid expressing CD8-CIMPR. Cells were fixed 24 hpt and stained with DAPI and an antibody recognizing EEA1. Fluorescence images of single confocal planes are shown: CD8-CIMPR, green; EEA1, magenta; nuclei, blue. Merged images show overlap between CD8-CIMPR and EEA1 pseudocolored

Journal of Cell Science • Supplementary information

white. Graph shows Manders coefficients for colocalization of CD8-CIMPR and EEA1 in cells expressing CD8-CIMPR for a representative experiment. The graph and statistical analysis are as in Fig. S2A. **, $p<0.01$; ****, $p<0.0001$. Similar results were obtained in three independent experiments. (**B**) As in (**A**) except cells were stained with an antibody recognizing TGN46 instead of EEA1, and merged images show overlap between CD8-CIMPR and TNG46. (**C**) HeLa control and PS1 KO cells were transfected with a plasmid expressing CD8-CIMPR and cotransfected with the empty control plasmid or a plasmid expressing FLAG-PS1. Cells were fixed 24 hpt and stained with DAPI and antibodies recognizing EEA1, CD8, and FLAG. Fluorescent images of single confocal planes are shown: CD8-CIMPR, green; EEA1, magenta; FLAG-PS1, red; nuclei, blue. Merged images show overlap between CD8-CIMPR and EEA1 pseudocolored white. Graph shows Manders coefficients for colocalization of CD8-CIMPR and EEA1 in cells expressing CD8-CIMPR (or in cells co-expressing CD8-CIMPR and FLAG-PS1 in the case of cells transfected with plasmid expressing wild-type or mutant PS1) for a representative experiment. The graph and statistical analysis are as in Fig. S2A. ***, $p<0.001$; ****, $p<0.0001$. Similar results were obtained in two independent experiments. (**D**) As in (**C**) except cells were stained with antibodies recognizing TGN46 and FLAG, and merged images show overlap between CD8-CIMPR and TGN46.

Journal of Cell Science • Supplementary information

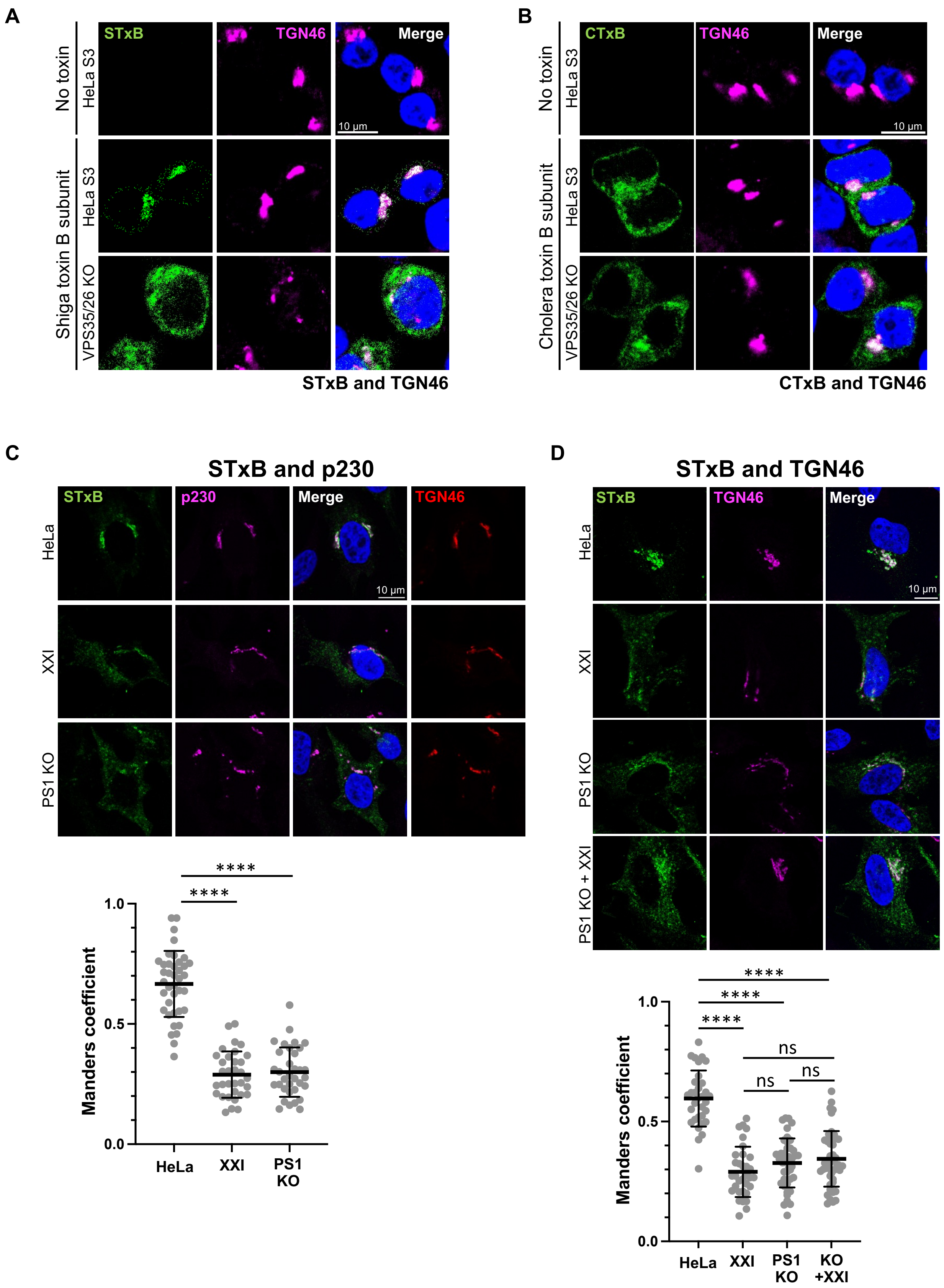
A
STxB
TGN46
Merge
No toxin
HeLa S3
10 µm
Shiga toxin B subunit
HeLa S3
VPS35/26 KO
STxB and TGN46
B
CTxB
TGN46
Merge
No toxin
HeLa S3
10 µm
Cholera toxin B subunit
HeLa S3
VPS35/26 KO
CTxB and TGN46
C
STxB and p230
STxB
p230
Merge
TGN46
HeLa
XXI
PS1 KO
10 µm
Manders coefficient
1.0
0.5
0.0
****
****
HeLa
XXI
PS1 KO
D
STxB and TGN46
STxB
TGN46
Merge
HeLa
XXI
PS1 KO
PS1 KO + XXI
10 µm
Manders coefficient
1.0
0.5
0.0
****
****
****
ns
ns
ns
HeLa
XXI
PS1 KO
KO +XXI

Fig. S4. Retromer knockout inhibits retrograde trafficking of shiga toxin but not cholera toxin. (**A**) HeLa S3 cells and VPS26/35 KO cells were incubated with or without 1 µg/ml fluorescent STxB. Cells were fixed 30 min after the treatment and stained with DAPI and an antibody recognizing TGN46. Fluorescent images of single confocal planes are shown: STxB, green; TGN46, magenta; nuclei, blue. Merged image shows overlap between STxB and TGN46 pseudocolored white. Similar results were obtained in two independent experiments. (**B**) As in (**A**) except cells were incubated with 1 µg/ml fluorescent CTxB. Merged images show overlap between CTxB and TGN46. Quantitation of the results shown in panels (**A**) and (**B**) is shown in Fig. 2A and B. (**C**) HeLa control and PS1 KO HeLa cells were treated with DMSO or XXI for 24 h and then incubated with 1 µg/ml fluorescent STxB. Cells were fixed 30 min after treatment and stained with DAPI and antibodies recognizing p230 and TGN46. Fluorescent images of single confocal planes are shown: STxB, green; p230, magenta; nuclei, blue; TGN46, red. Merged images show overlap between STxB and p230 pseudocolored white. Graph shows Manders coefficients for colocalization between STxB and p230 in cells containing detectable toxin in a representative experiment. The graph and statistical analysis are as in Fig. S2A. ****, p<0.0001. Similar results were obtained in two independent experiments. (**D**) HeLa control and PS1 KO HeLa cells were treated with DMSO or XXI for 24 h and then incubated with 1 µg/ml fluorescent STxB. Cells were fixed 30 min after treatment and stained with DAPI and an antibody recognizing TGN46. Fluorescent images of single confocal planes are shown: STxB, green; TGN46, magenta; nuclei, blue. Merged images show overlap between STxB and TGN46 pseudocolored white. Graph shows Manders coefficients for colocalization between STxB and TGN46 in cells containing detectable toxin for a representative experiment. The graph and statistical analysis are as in Fig. S2A. ****, p<0.0001; ns, not significant. Similar results were obtained in two independent experiments.

Journal of Cell Science • Supplementary information

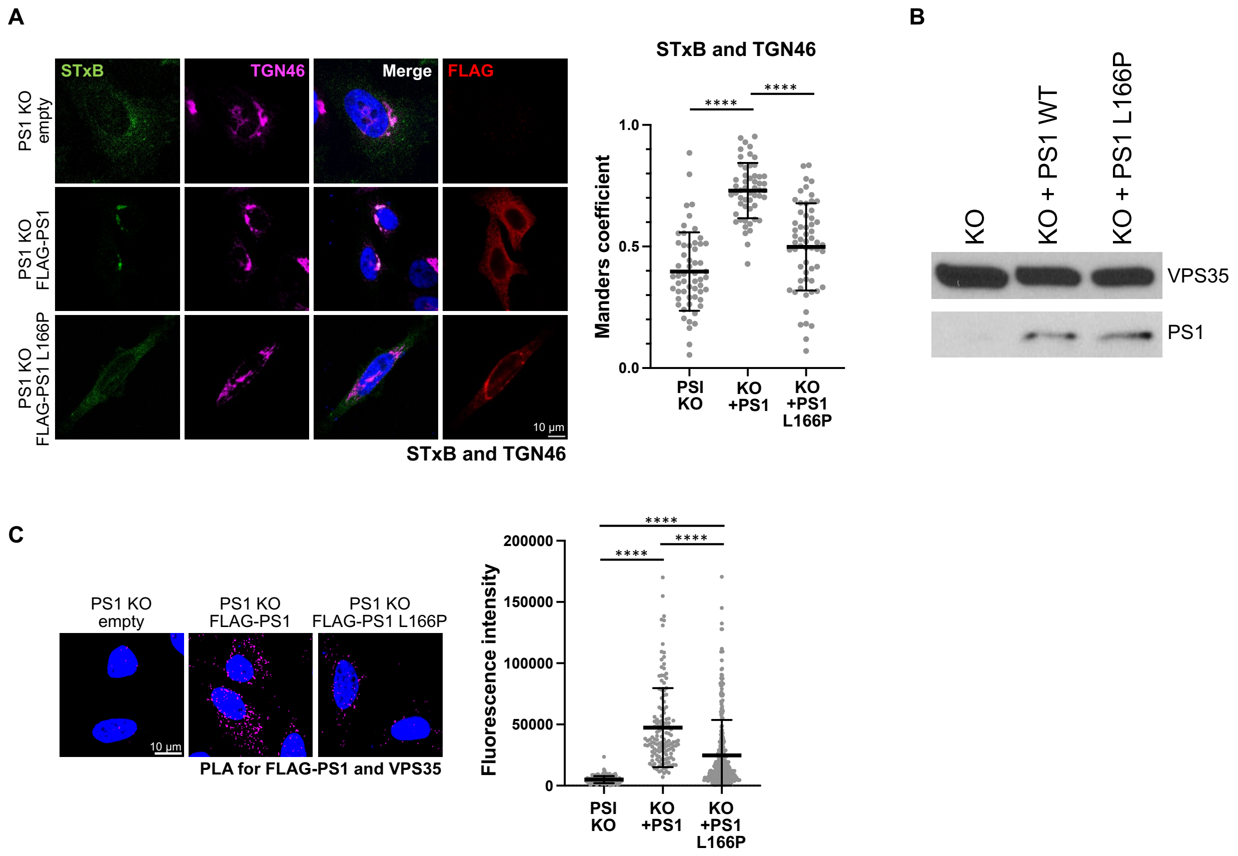

Fig. S5. The L166P PS1 mutant does not support trafficking of shiga toxin and shows decreased interaction with retromer.

(A) PS1 KO HeLa cells were transfected with the empty control plasmid or a plasmid expressing wild-type FLAG-PS1 or FLAG-PS1 L166P. 24 hpt, cells were incubated with 1 µg/ml fluorescent STxB. Cells were fixed 30 min later and stained with DAPI and antibodies recognizing TGN46 and FLAG. Fluorescent images of single confocal planes are shown: STxB, green; TGN46, magenta; FLAG-PS1, red; nuclei, blue. Merged images show overlap between STxB and TGN46 pseudocolored white. Graph shows Manders coefficients for colocalization of STxB and TGN46 in cells expressing STxB (or in cells co-expressing STxB and FLAG-PS1 or FLAG-PS1 L166P in the case of cells transfected with plasmids expressing wild-type or mutant PS1 [KO + FLAG-PS1 or KO + FLAG-PS1 L166P]). The graph and statistical analysis are as in Fig. S2A. ****, p<0.0001. Similar results were obtained in two independent experiments. (**B**) Extracts were prepared from PS1 KO HeLa cells transfected with the empty control plasmid or a plasmid expressing wild-type FLAG-PS1 (WT) or FLAG-PS1 L166P for 24 h. Cell extracts were

Journal of Cell Science • Supplementary information

subjected to western blot analysis using antibodies recognizing endogenous VPS35 or FLAG. (**C**) PS1 KO HeLa cells were transfected with the empty control plasmid or a plasmid expressing FLAG-PS1 or FLAG-PS1 L166P. Cells were fixed 24 hpt. PLA was performed with antibodies recognizing FLAG and VPS35. Images show single confocal planes. PLA signals are magenta; nuclei are stained blue with DAPI. The fluorescence of PLA signals was determined from multiple images. Each dot represents PLA fluorescence intensity of an individual cell (n>140) for a representative experiment. The statistical analysis is as in Fig. S2A. ****$p<0.0001$. Similar results were obtained in two independent experiments.

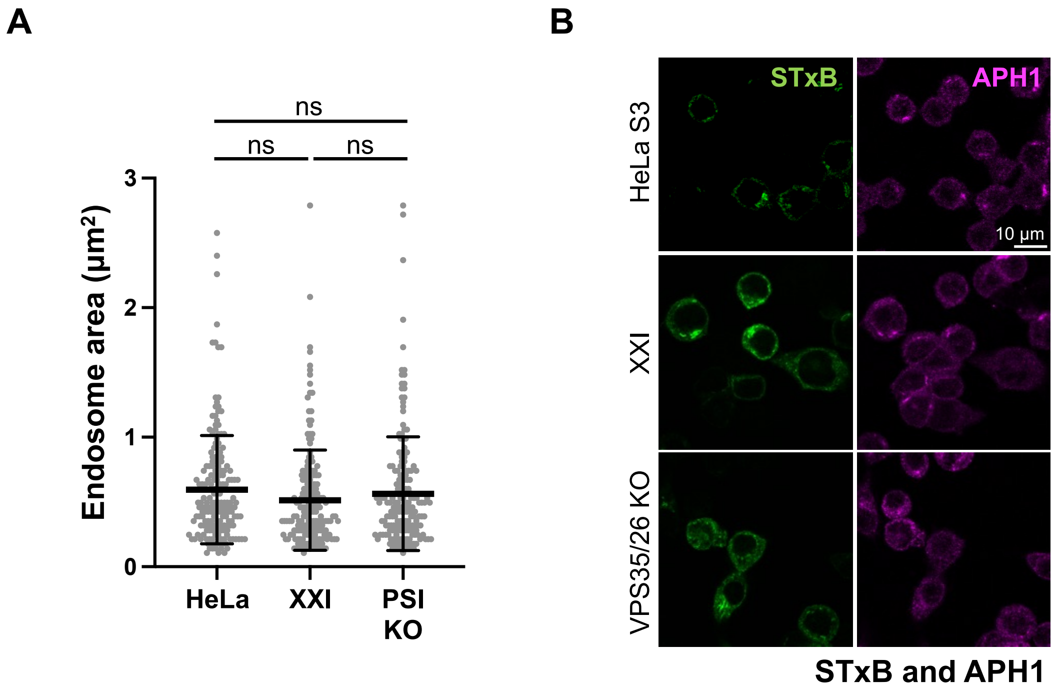

Fig. S6. Analysis of endosomal size and cell heterogeneity.

(**A**) Images as in Figure 3B were analyzed. Endosome area (i.e. EEA1 staining) from multiple images was determined. Each dot represents an individual endosome (n>150). The statistical analysis is as in Fig. S2A. ns, not significant. Similar results were obtained in three independent experiments. (**B**) HeLa S3 and VPS26/35 KO cells were treated with DMSO or 1 µM XXI for 24 h and then incubated with 1 µg/ml fluorescent STxB for 30 min. Cells were fixed and stained with DAPI and an antibody recognizing APH1. Fluorescent confocal images were captured to examine distribution of STxB and APH1. These images are similar as in Fig. 6C, but lower magnification to show patterns of APH1 and STxB staining in a larger number of cells.

Journal of Cell Science • Supplementary information

Fig. 3A

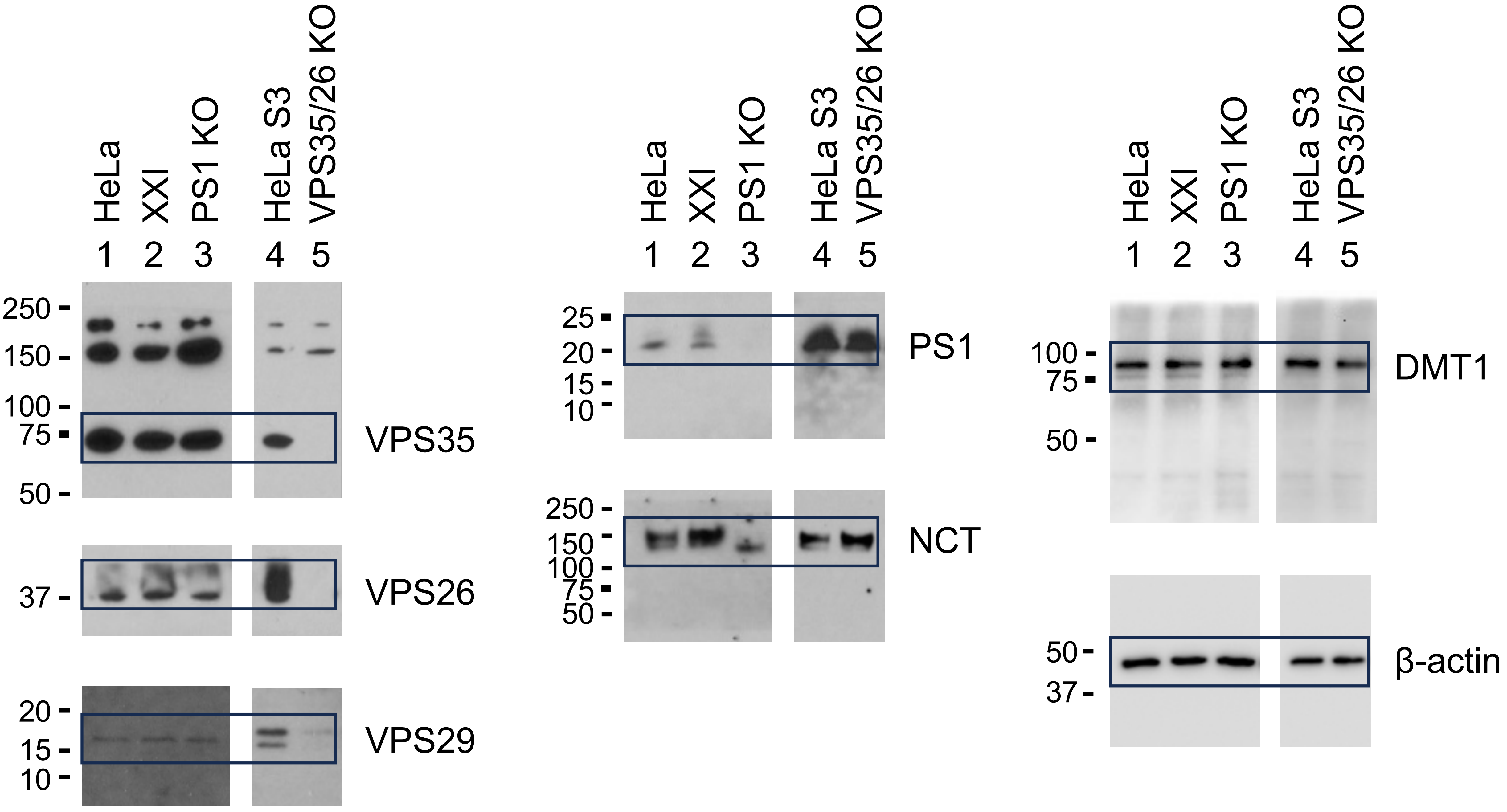

Journal of Cell Science • Supplementary information

Fig. 4A

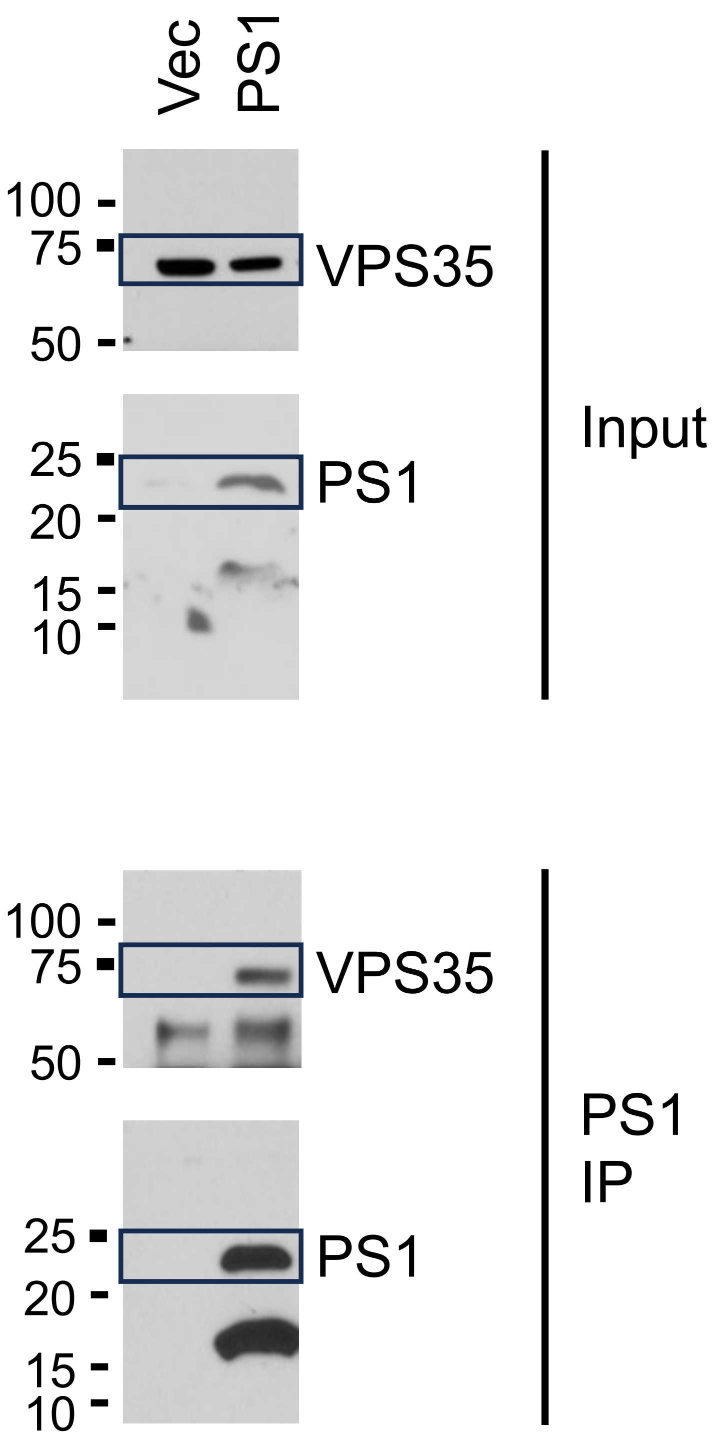

Fig. 4B

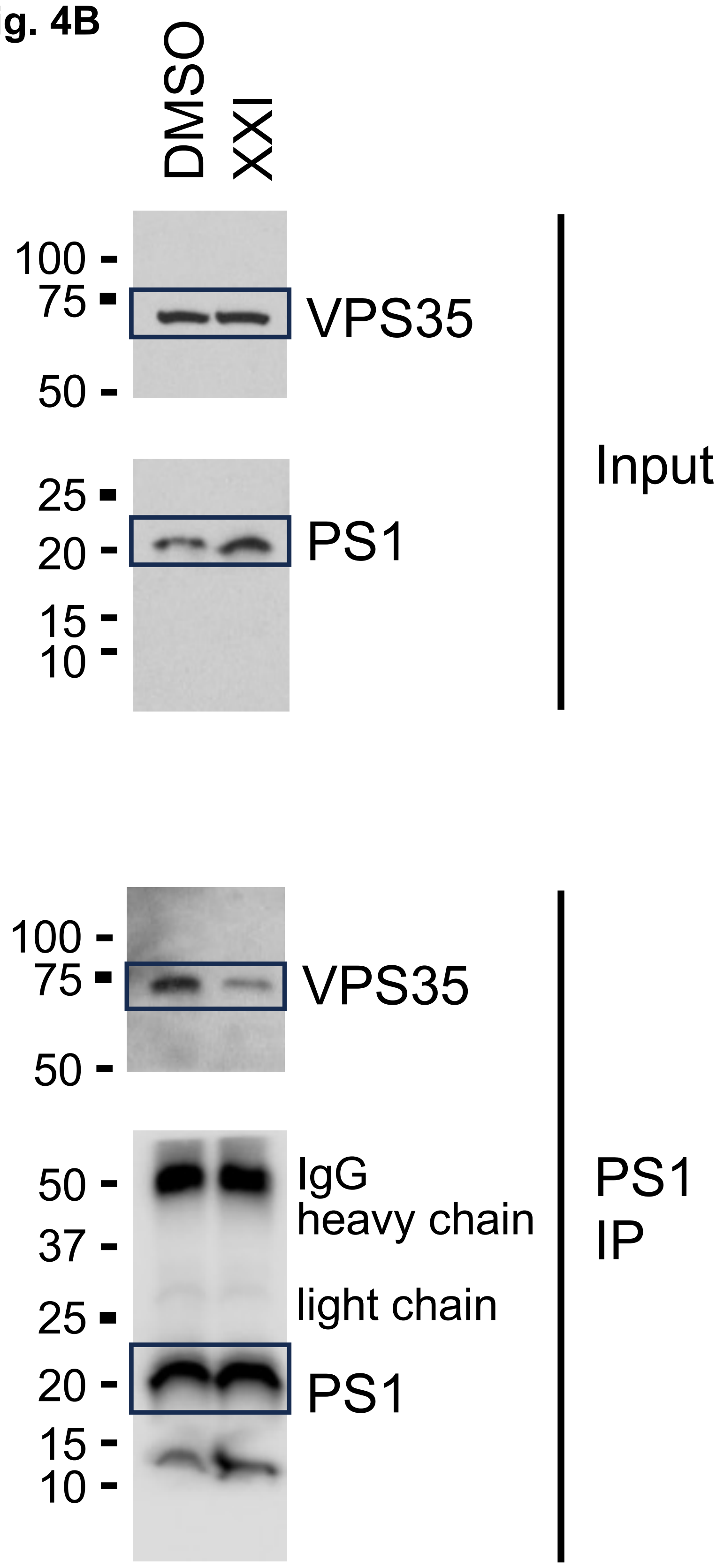

Journal of Cell Science • Supplementary information

Fig. 5D

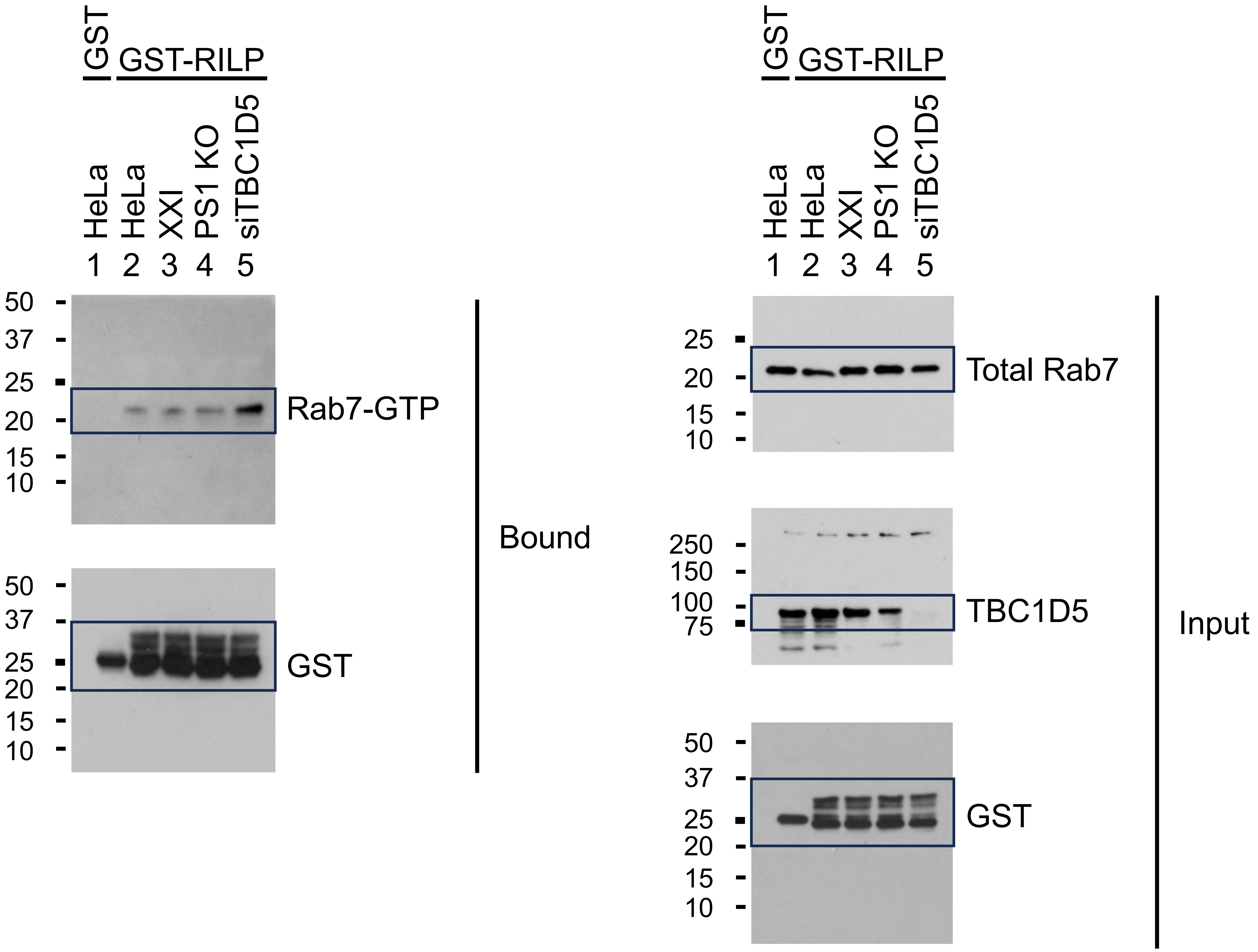

GST
GST-RILP
HeLa
HeLa
XXI
PS1 KO
siTBC1D5
1 2 3 4 5
50
37
25
20
15
10
Rab7-GTP
GST
Bound
Total Rab7
250
150
100
75
TBC1D5
GST
Input

Fig. 6A

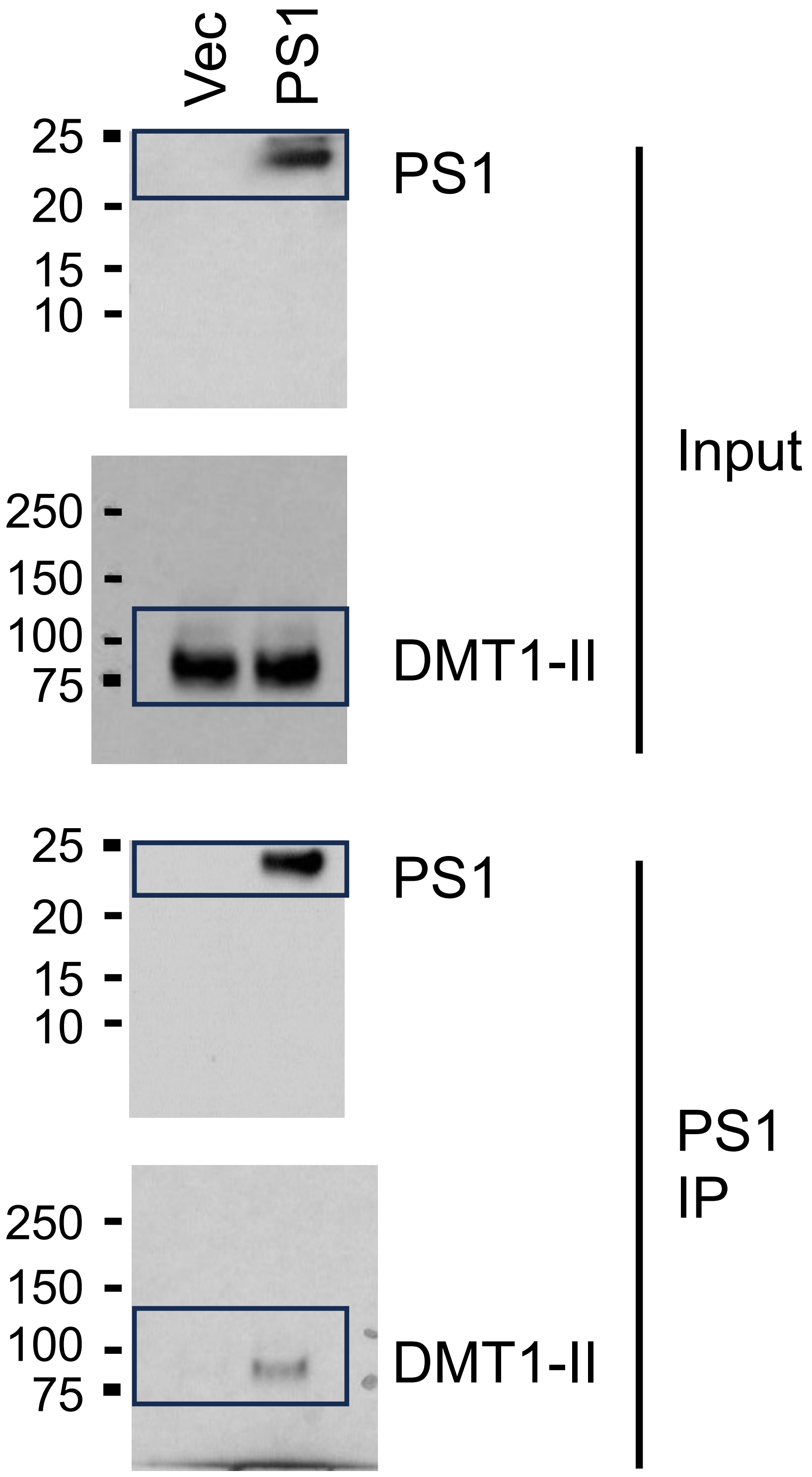

Fig. 6B

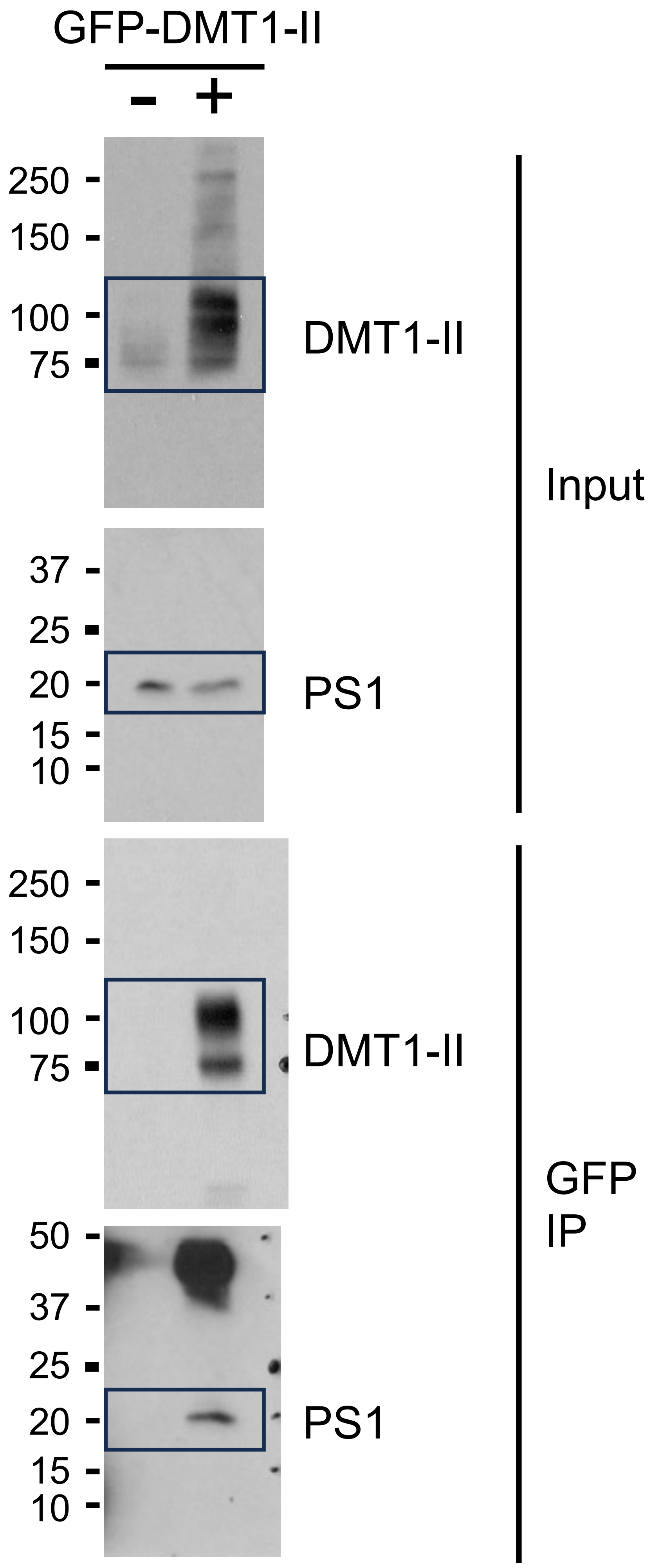

Journal of Cell Science • Supplementary information

Fig. 6C

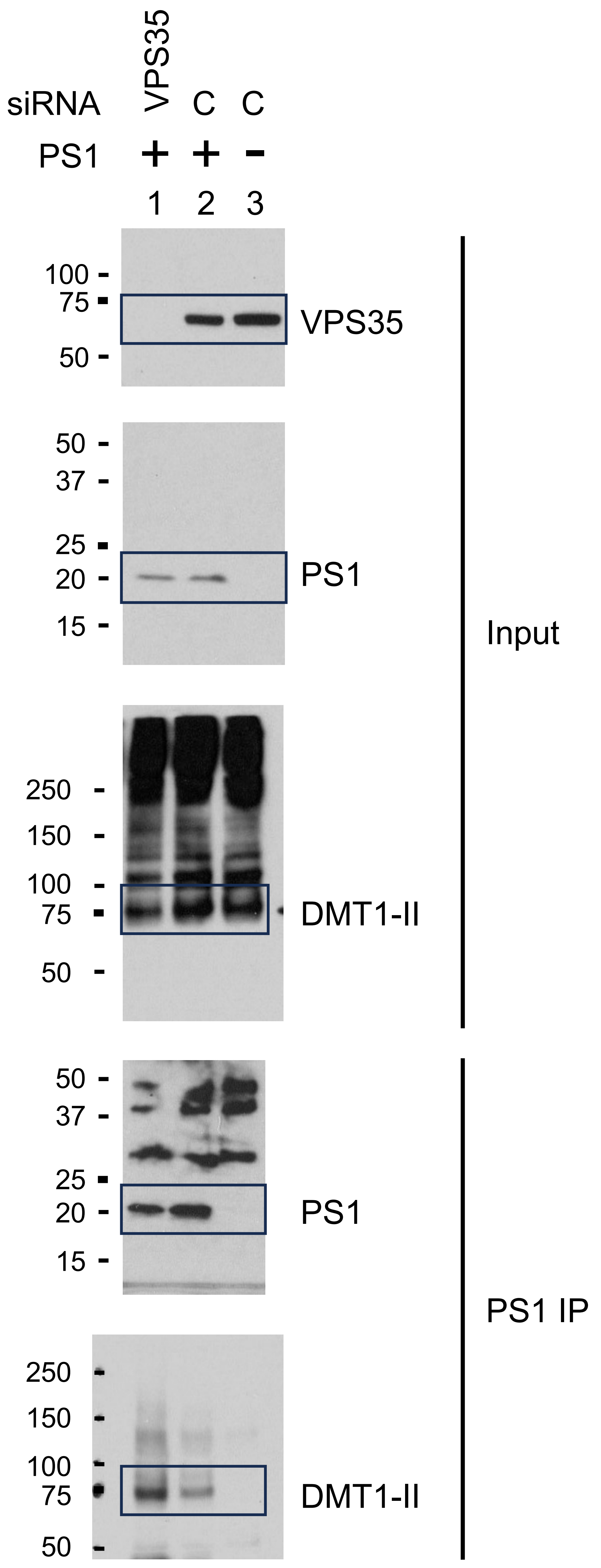

Fig. S5B

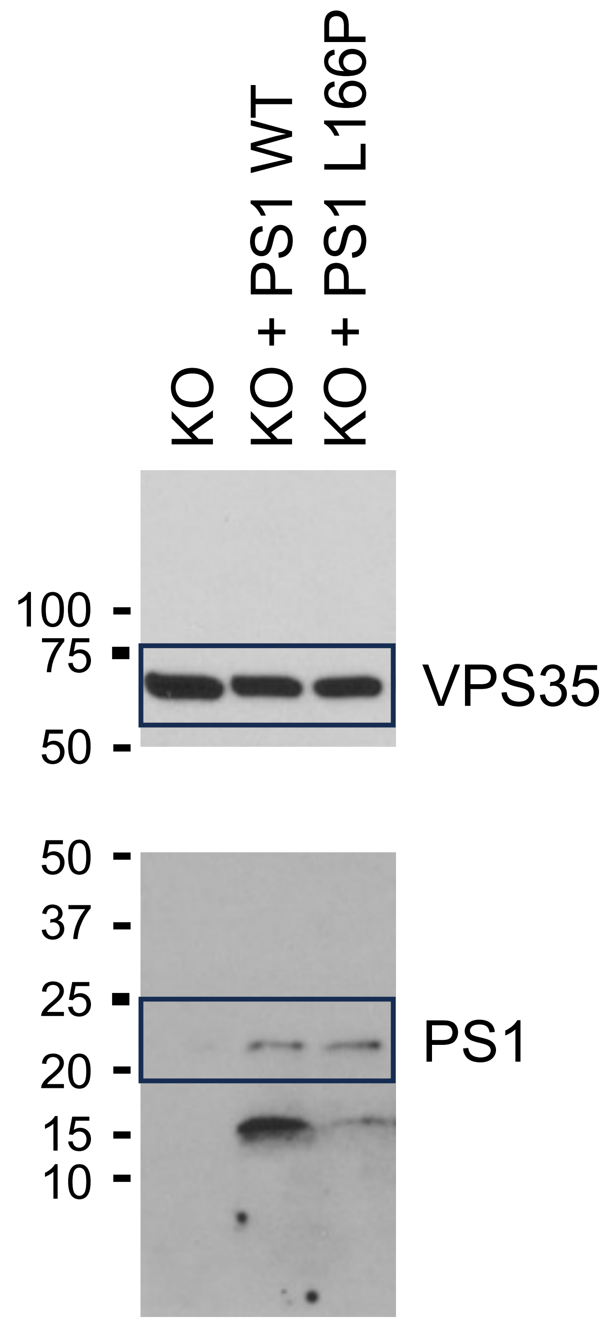

Journal of Cell Science • Supplementary information

Fig. S7. Blot transparency.

This and the following four pages show original images of the western blot data. Typically, we cut the membranes prior to probing so that we could probe the same blot with multiple different antibodies to detect proteins that display different mobilities. The black outlines indicate the portion of the image that is included in the figures in the manuscript.

Journal of Cell Science • Supplementary information

Table S1. List of inhibitors used

**Inhibitors**	Source	Catalog no.
XXI	Sigma	565790
Shiga toxin B subunit	Sigma	SML0562
Cholera toxin B subunit	Thermo Fisher Scientific	C34775

Table S2. List of antibodies used

**Antigen**	Source	Catalog no.	Species	Application	Dilution
EEA1	Cell Signaling	2411	Rabbit	IF	1:100
EEA1	BD	610457	Mouse	IF	1:100
TGN46	Abcam	ab50595	Rabbit	IF	1:200
p230	BD	611280	Mouse	IF	1:200
CD8	AdipoGen	Anc153-020	Mouse	IF, PLA	1:400
VPS35	Abcam	ab157220	Rabbit	PLA, WB	1:200, 1:1000 for WB
VPS35	Abcam	ab57632	Mouse	PLA, WB	1:200, 1:1000 for WB
VPS26	Abcam	ab23892	Rabbit	IF, WB	1:200, 1:1000 for WB
VPS29	Abcam	ab236796	Rabbit	WB	1:1000
PS1	Cell Signaling	5643	Rabbit	WB	1:1000
NCT	Santa Cruz	sc-376513	Mouse	WB	1:1000
APH1	Thermo Fisher	PA1-2010	Rabbit	IF, PLA	1:50
DMT1	Abcam	ab55735	Mouse	WB	1:1000
GFP	Santa Cruz	sc-9996	Mouse	PLA	1:100
GFP-trap	chromotek	Gtma-20	Alpaca	IP	25 µL/sample
Rab7	Cell Signaling	9367	Rabbit	WB	1:1000
GST-HRP	Santa Cruz	sc-138	Mouse	WB	1:2000
FLAG	Thermo Fisher	MA1-142	Rat	IF	1:500
TBC1D5	Abcam	Ab203896	Rabbit	WB	1:1000
pan actin	Cell Signaling	4968	Rabbit	WB	1:2000

Table S3. List of secondary antibodies used

**Secondary antibodies (Thermo Fisher Scientific)**	Catalog#
Donkey anti-Mouse IgG (H+L) Highly Cross-Adsorbed Secondary Antibody, Alexa Fluor™ 647	A31571
Goat anti-Rabbit IgG (H+L) Highly Cross-Adsorbed Secondary Antibody, Alexa Fluor™ 647	A21245
Goat anti-Rat IgG (H+L) Cross-Adsorbed Secondary Antibody, Alexa Fluor™ 647	A21247
Donkey anti-Rabbit IgG (H+L) Highly Cross-Adsorbed Secondary Antibody, Alexa Fluor™ 568	A10042
Donkey anti-Mouse IgG (H+L) Highly Cross-Adsorbed Secondary Antibody, Alexa Fluor™ 488	A21202
Donkey anti-Rabbit IgG (H+L) Highly Cross-Adsorbed Secondary Antibody, Alexa Fluor™ 488	A21206

Journal of Cell Science • Supplementary information
